# Global perspective of ecological risk of plastic pollution on soil microbial communities

**DOI:** 10.3389/fmicb.2024.1468592

**Published:** 2024-10-09

**Authors:** Bing Yang, Lin Wu, Wanju Feng, Qi Lin

**Affiliations:** Sichuan Academy of Giant Panda, Chengdu, China

**Keywords:** ecological risks, micro-plastics, nano-plastics, plastic residues, soil microorganism, terrestrial ecosystem

## Abstract

**Introduction:**

The impacts of plastic pollution on soil ecosystems have emerged as a significant global environmental concern. The progress in understanding how plastic pollution affects soil microbial communities and ecological functions is essential for addressing this issue effectively.

**Methods:**

A bibliometric analysis was conducted on the literature from the Web of Science Core Collection database to offer valuable insights into the dynamics and trends in this field.

**Results:**

To date, the effects of plastic residues on soil enzymatic activities, microbial biomass, respiration rate, community diversity and functions have been examined, whereas the effects of plastic pollution on soil microbes are still controversial.

**Discussion:**

To include a comprehensive examination of the combined effects of plastic residue properties (Type, element composition, size and age), soil properties (soil texture, pH) at environmentally relevant concentrations with various exposure durations under field conditions in future studies is crucial for a holistic understanding of the impact of plastic pollution on soil ecosystems. Risk assessment of plastic pollution, particularly for nanoplasctics, from the perspective of soil food web and ecosystem multifunctioning is also needed. By addressing critical knowledge gaps, scholars can play a pivotal role in developing strategies to mitigate the ecological risks posed by plastic pollution on soil microorganisms.

## 1 Introduction

The significance of soil microbial communities extends beyond their involvement in essential biogeochemical cycles (Bardgett and van der Putten, 2014), as they also play a crucial role in shaping the well-being of plants, animals, and humans (Romdhane et al., 2022). Prior research has suggested that a decline in microbial diversity and the simplification of soil community composition are likely to have detrimental effects on the ability of terrestrial ecosystems to provide vital services such as climate regulation, soil fertility, and food and fiber production (Delgado-Baquerizo et al., 2016; Wagg et al., 2014). The significance of microorganisms in ecosystem functioning has led to increased global interest in the impacts of anthropogenic activities on soil microbial responses (Banerjee et al., 2018; Bardgett and van der Putten, 2014). Previous research has highlighted soil physicochemical properties, including soil water content and organic carbon availability (Drenovsky et al., 2004) and pH (Bahram et al., 2018; Fierer and Jackson, 2006), as key determinants of soil microbial community.

Plastic materials are widely used in the industrial engineering, agriculture and medical industry and other fields due to their high versatility, stability, light weight, and low production costs (Fuller and Gautam, 2016; Ya et al., 2021). However, limitations of recycling technology and long durability result in plastic accumulation in natural environment, especially soil (Boots et al., 2019; Ya et al., 2021). Under long-term photo-oxidative, thermal, ozone-induced, mechanic-chemical, catalytic and biological processes, plastic debris can be decomposed into micro-plastics (Thompson et al., 2004), referring to plastic debris with a size less than 5 mm, are an emerging contaminant of soil (Boots et al., 2019; de Souza Machado et al., 2018a; Ding L. et al., 2022; Guo et al., 2020; Li H. Z. et al., 2021; Wang Q. et al., 2021; Yang B. et al., 2022). Previous studies have demonstrated that micro-plastics entre soils through multiple pathways (Guo et al., 2020; Kumar et al., 2020; Yang B. et al., 2022), such as compost and sewage sludge application (Corradini et al., 2019; Schell et al., 2022; van den Berg et al., 2020; Weithmann et al., 2018; Yang M. et al., 2021; Yang J. et al., 2021; Zhang J. et al., 2023; Zhang L. et al., 2020), residual plastic mulching (Huang et al., 2020; Khalid et al., 2023; Qi R. et al., 2020; Qi et al., 2018; Steinmetz et al., 2022; Zhou B. et al., 2020) and micro-plastic seed film-coating (Accinelli et al., 2019), atmospheric fallout (Allen et al., 2019; Bergmann et al., 2019; Brahney et al., 2020; Corradini et al., 2019; Nizzetto et al., 2016; Scheurer and Bigalke, 2018), wastewater irrigation (He et al., 2018; Perez-Reveron et al., 2022), wind erosion (Bullard et al., 2021; Rezaei et al., 2022; Rezaei et al., 2019), and tire wear (Ding J. et al., 2022).

Plastic pollution has emerged as a critical environmental issue with far-reaching impacts on soil ecosystems, particularly on microbial communities that play essential roles in nutrient cycling and soil health. Recent studies have demonstrated that microplastics can alter the physical and chemical properties of soil (Boots et al., 2019; de Souza Machado et al., 2018b; Feng et al., 2022; Lozano et al., 2021a; Qi Y. et al., 2020; Wang et al., 2020), leading to changes in microbial diversity, abundance, and function. Additionally, microplastics can serve as vectors for harmful chemicals and pathogens, thereby influencing microbial community structure and resilience. Studies have reported that micro-plastics and/or nano-plastics can be carriers for other soil pollutants (Rillig et al., 2019a; Zhang S. et al., 2020), absorbing and transporting harmful organic pollutants (Huffer et al., 2019; Li et al., 2016; Zhang B. et al., 2020; Zhang et al., 2021), antibiotics (Li et al., 2018; Zhu D. et al., 2022; Zhu F. et al., 2022), heavy metals (Abbasi et al., 2020; Zhang B. et al., 2020), xenobiotics (Hummel et al., 2021) and act as a vector for bacterial disease (Beloe et al., 2022). Furthermore, the degradation of plastics introduces a variety of chemical compounds into the soil, which can be metabolized by certain microbes, leading to shifts in community composition. In addition, micro-plastics provide additional habitats for many microbial communities in the environment (Li et al., 2023; Rüthi et al., 2020; Wang et al., 2022; Wang et al., 2023; Yang Y. et al., 2022; Zhang et al., 2019; Zhu D. et al., 2022; Zhu F. et al., 2022). Finally, micro-plastics affect plant (Rillig et al., 2019b), which in turn mediate soil microbial community through alterations of rhizodoposition as well as litter quality and quantity. In the last decades, the impacts of plastic contamination on soil microbial community have been extensively studied (Rillig et al., 2019a). The development trends indicate an increasing concern over the long-term ecological consequences of plastic pollution, with research focusing on understanding the mechanisms of microbial adaptation and the potential for bioremediation. Addressing these impacts is crucial for preserving soil health and ensuring sustainable agricultural practices, highlighting the need for ongoing research and policy interventions. However, to our knowledge, no report has revealed the status and development trends of impacts of plastic pollution on soil microbial community using data mining yet.

The subfield of scientometrics known as bibliometric analysis employs computer technology and statistical methods to quantitatively assess the current state of research, areas of high interest, and emerging trends within a particular research domain (Li J. et al., 2021; Zhang and Chen, 2020). Furthermore, bibliometric analysis has gained widespread recognition as a valuable tool for guiding novice researchers (Fu et al., 2010), offering valuable insights into the development and evolutionary patterns of disciplines, collaborative efforts, and prospective directions. Since the Science Citation Index Expanded (SCI-E) database provides comprehensive coverage of the most important and influential research results from all over the world (Zhang and Chen, 2020), we do a bibliometric collection based on the SCI-E database in the “Web of Science Core Collection.” Considering the unique advantages and drawbacks of bibliometric analysis tools, such as Bibexcel, Bibliometrix (Aria and Cuccurullo, 2017), BiblioMaps (Grauwin and Sperano, 2018), CiteSpace (Chen, 2006), CitNetExplore (Thor et al., 2016), SciMAT (Cobo et al., 2012), Sci2 Tool (Börner, 2011), VOSviewer (van Eck and Waltman, 2010), we utilized three commonly used bibliometric analysis software simultaneously.

This study aims to provide a comprehensive overview of the Performance, critical knowledge gaps, and development directions of studying plastic-soil-microorganism interaction through bibliometric analysis, and to offer insights and suggestions for the future development of this field.

## 2 Materials and methods

### 2.1 Data collection

A comprehensive literature search on the Web of Science Core Collection (WoSCC) database^1^ on January 1, 2024. The search formula was defined as follows: ((TS = (microplastic* or nanoplasctic*)) AND TS = (soil microbial community or soil bacterial community or soil fungal community)) AND LA = (English), and the type of documents is set to “articles “OR “review” (Supplementary Figure S1). A total of 452 English peer-reviewed publications were retrieved with selected information (including title, keywords, abstract, introduction, author information, journals, citation, and institutional affiliation).

### 2.2 Data analysis

In the present study, three analytical software tools were simultaneously used. Firstly, the overview, consisting the main information, annual scientific production, sources, authors, affiliations, countries, most globally cited documents, most frequent words, word dynamics, clustering by coupling were analyzed using the Bibliometrix, which contains the more extensive set of techniques and are suitable for practitioners through Biblioshiny (Moral-Muñoz et al., 2020). Additionally, the visualization of co-authorship networks and the co-occurrence networks of keywords were implemented using VOSviewer version 1.6.18 (Centre for Science and Technology Studies, Leiden University, The Netherlands). The collaborative relationship between authors sharing more than two publications was determined by co-authorship analysis using VOSviewer. Finally, keyword clustering network analysis, co-citation reference analysis and the burst analysis were performed using the CiteSpace version 6.2. R6 (a keyword clustering map was generated using the log-likelihood ratio algorithm (LLS) after eliminating relevant subject words).

## 3 Results

### 3.1 Publication outputs

**Figure 1:**
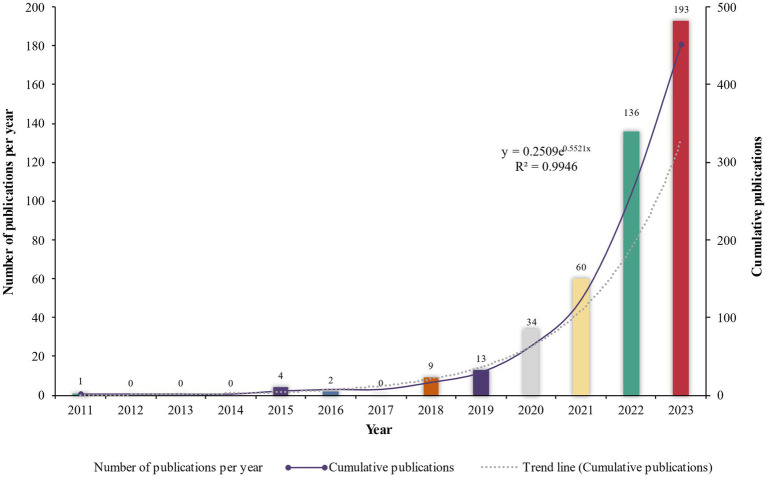
Distribution of annual and cumulative scientific publications addressing potential impacts of plastics pollution on soil microbial communities gained from the Web of Science (WoS) core collection from 2011to 2023.

As shown in Figure 1, the annual number of articles on potential ecological risks of plastic contaminate showed an overall upward trend during 2011–2023. From 2011 to 2017, fewer papers were published, whereas the growth rate of published papers has accelerated since 2018, and the number of papers published in 2023 has risen to 193. Further analysis showed that the cumulative publications experienced an exponential growth ( $y=0.2509e^{0.5521x},R^{2}=0.9619$ ) during 2011–2023 (Figure 1). It shows that studying on ecological risks of plastic containment on soil microorganisms has gradually increased and received more and more attention in recent years.

### 3.2 Countries/regions and institutions

**Figure 2:**
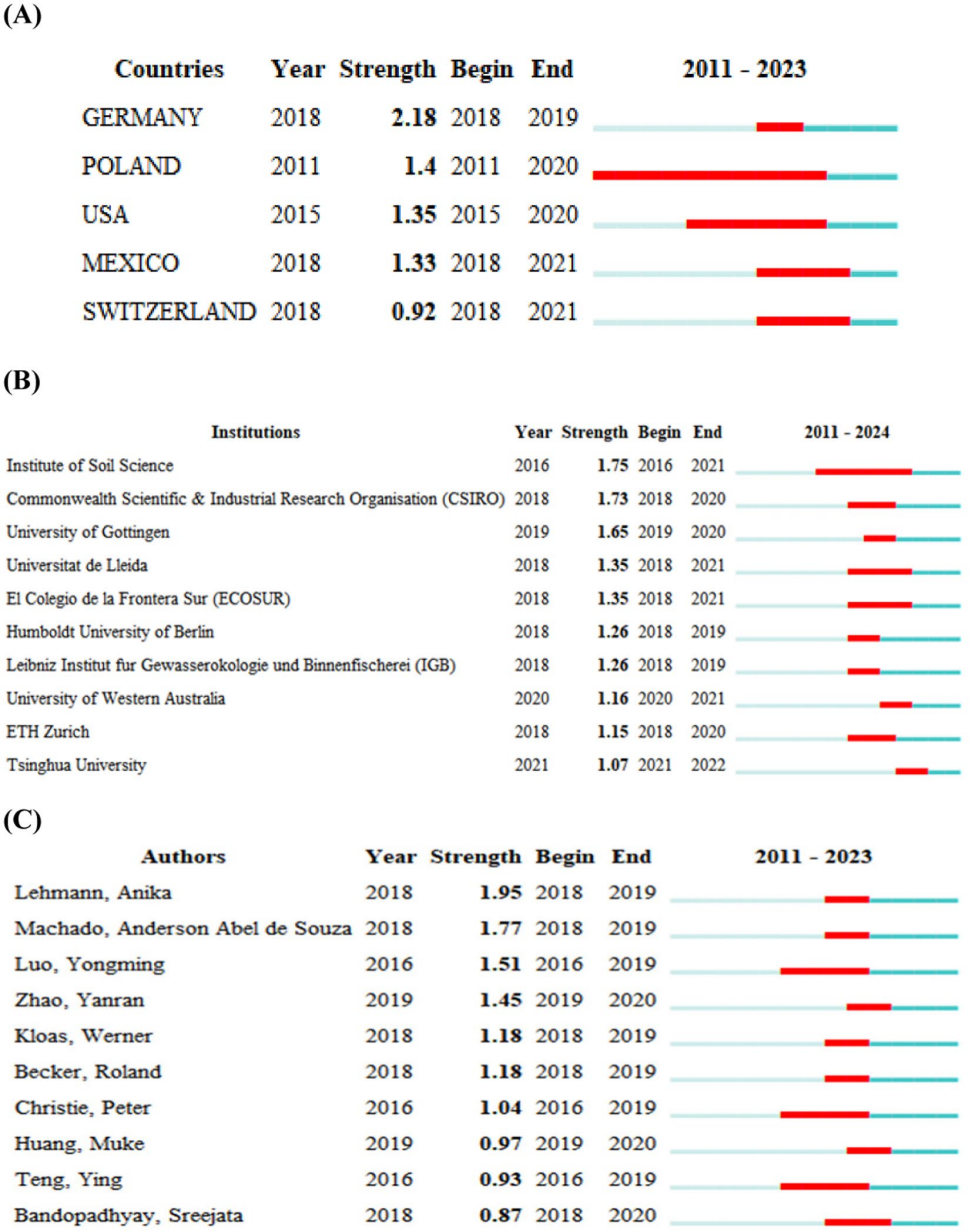
Top five countries **(A)**, top ten institutions **(B)** and top ten authors **(C)** with strongest burst.

From the country level, China ranked the first productive country on the impacts of plastic pollution on soil microbial communities, with 322 papers published, accounting for 71.24% of the total publications. This was followed by Germany and the United States, with 46 and 42 publications, respectively. Further examination showed that the publications of the Germany, Poland, United States, Mexico, and Switzerland has exploded recently (Figure 2A). Among which, the strongest burst intensity was found in Germany, with a burst intensity of 2.18 and a burst period of 2018–2019, whereas the longest lasting burst time was found in Poland, with a period spanning from 2011 to 2020. From the institutional level (Table 1), Chinese Academy of Sciences has the highest number of publications, with 67 articles, ranking the first. This was followed by University of Chinese Academy of Sciences, with 25 papers. During 2016–2021, the Institute of Soil Science, Chinese Academy of Sciences experienced a surge in publications, with a burst intensity of 1.75, and the longest burst time, whereas the Tsinghua University has a relatively late burst time in comparison with other institutions, from 2021 to 2022 (Figure 2B).

**Table 1:** Top 15 most productive institutions of studying the ecological risks of plastics pollution on soil microbial community.

Rank	Institutions	Publications	Citations	Country
1	Chinese Academy of Sciences	67	3,004	China
2	China Agricultural University	25	1,237	China
3	University of Chinese Academy of Sciences	24	1,100	China
4	Nankai University	23	1,105	China
5	Freie Universität Berlin	22	2,187	Germany
6	Northwest A&F University	21	566	China
7	Ministry of Agriculture and Rural Affairs of the People’s Republic of China	20	454	China
8	Nanjing University	19	698	China
9	Peking University	15	945	China
10	Bangor University	14	923	United Kingdom
11	Wageningen University & Research	13	1,115	Netherland
12	Berlin-Brandenburg Institute of Advanced Biodiversity Research	12	1762	Germany
13	Beijing Academy of Agriculture and Forestry Sciences	11	202	China
14	Chinese Research Academy of Environmental Sciences	11	265	China
15	Chinese Academy of Agricultural Sciences	10	468	China

**Figure 3:**
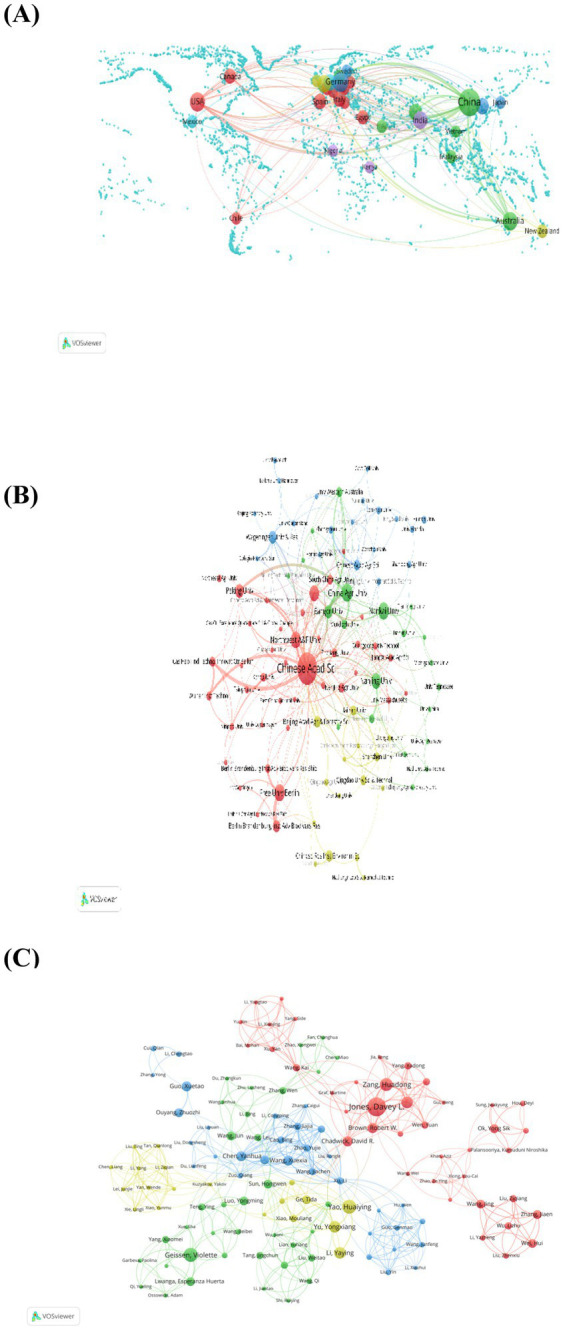
Collaboration network between countries **(A)**, core institutions **(B)**, and core authors **(C)**. In the maps, the size of the circle represents of the number of documents, and the thickness of the lines depicts the strength of the association. In general, the larger the nodes, the stronger the collaboration between country, institution, and authors.

Additionally, there were strong close cooperation across some countries (Figure 3A) and institutions (Figure 3B). China is the most willing to cooperate with other countries. Notably, China and the United States have the closest cooperative relationship. Chinese Academy of Sciences has the strongest willingness to cooperate with other institutions, whereas the closest cooperative relationship was observed between Free University of Berlin and Berlin-Brandenburg Institute of Advanced Biodiversity Research. The collaborations between China Agricultural University and Bangor University were also frequently.

### 3.3 Authors

A total of 23 authors published at least five articles during the examined period (Table 2). Specifically, professor Davey L. Jones from Bangor University was the most productive author in terms of published articles. He published 13 papers with 881 citations, followed by professor Geissen Violette from Wageningen University & Research and professor Yao Huaiying from Institute of Urban Environment, Chinese Academy of Sciences, respectively. Both the afore-mentioned two authors published nine articles, whereas the citations were 1,027 and 242, respectively. The other authors published no less than five articles were Huadong Zang and Jie Zhou from China Agricultural University, Yaying Li from Institute of Urban Environment, Chinese Academy of Sciences, Yongxiang Yu from Wuhan Institute of Technology, Robert W. Brown and David R. Chadwick from Bangor University, Xuetao Guo and Zhuozhi Ouyang from Northwest A&F University, Yanhua Chen, Xuexia Wang, and Zuoyuan Zou from Beijing Academy of Agriculture and Forestry Sciences, Tida Ge from Ningbo University, Yongming Luo from Institute of Soil Science, Chinese Academy of Sciences, Esperanza Huerta Lwanga from Wageningen University & Research, Yong Sik Ok from Korea University, Hongwen Sun from Nankai University, as well as Jing Wang, Jun Wang, Hui Wei and Jiaen Zhang from South China Agricultural University (Table 2). Burst analysis showed that the publication number of Anika Lehmann has increased explosively during 2018–2019, with the first publication in 2018, and the publication number of Yongming Luo, Peter Christie, Ying Teng showed a steady increase during 2016–2019 (Figure 2C).

**Table 2:** Top authors with least five publications related to ecological risks of plastics pollution on soil microbial community.

Rank	Author	Publications	Links	Total link strength	Citations	Country/Region	Institution
1	Jone, Davey L.	13	16	55	881	United Kingdom	Bangor University
2	Geissen, Violette	9	12	24	1,027	Netherlands	Wageningen University & Research
3	Yao, Huaiying	9	8	27	242	China	Institute of Urban Environment, CAS
4	Zang, Huadong	8	8	27	449	China	China Agricultural University
5	Li, Yaying	7	4	15	187	China	Institute of Urban Environment, CAS
6	Yu, Yongxiang	7	8	25	104	China	Wuhan Institute of Technology
7	Zhou, Jie	7	10	36	432	China	China Agricultural University
8	Brown, Robert W.	6	14	30	91	United Kingdom	Bangor University
9	Chadwick, David R.	6	16	27	243	United Kingdom	Bangor University
10	Chen, Yanhua	6	17	43	75	China	Beijing Academy of Agriculture and Forestry Sciences
11	Guo, Xuetao	6	3	7	106	China	Northwest A&F University
12	Wang, Xuexia	6	12	38	94	China	Beijing Academy of Agriculture and Forestry Sciences
13	Ge, Tida	5	9	24	100	China	Ningbo University
14	Luo, Yongming	5	6	8	994	China	Institute of Soil Science, CAS
15	Lwanga, Esperanza Huerta	5	8	14	1,004	Netherlands	Wageningen University & Research
16	Ok, Yong Sik	5	5	12	343	Korea	Korea University
17	Ouyang, Zhuozhi	5	2	6	106	China	Northwest A&F University
18	Sun, Hongwen	5	10	12	199	China	Nankai University
19	Wang, Jing	5	12	26	23	China	South China Agricultural University
20	Wang, Jun	5	8	12	297	China	South China Agricultural University
21	Wei, Hui	5	6	23	42	China	South China Agricultural University
22	Zhang, Jiaen	5	6	23	42	China	South China Agricultural University
23	Zou, Guoyuan	5	16	43	29	China	Beijing Academy of Agriculture and Forestry Sciences

CAS, Chinese Academy of Sciences.

As presented in co-author network, several author groups work together closely and have significant links to other groups, including the clustering of an important collaborative team with Davey L. Jones and Geissen Violette as the core (Figure 3C). Davey L. Jones established extensive collaborations with other authors, as evidenced by the highest number of linkages. Among which, the closest partnership was found to that between him and Huadong Zang. Besides, there are scientific groups with significant scientific outputs but limited levels of cooperation with other groups among the most influential authors in this field. For instance, the collaborative team with Huaiying Yao as the core, which is at the edge of the entire network map, is less collaborative in intensity than those located near the center of the map.

### 3.4 Journals

**Figure 4:**
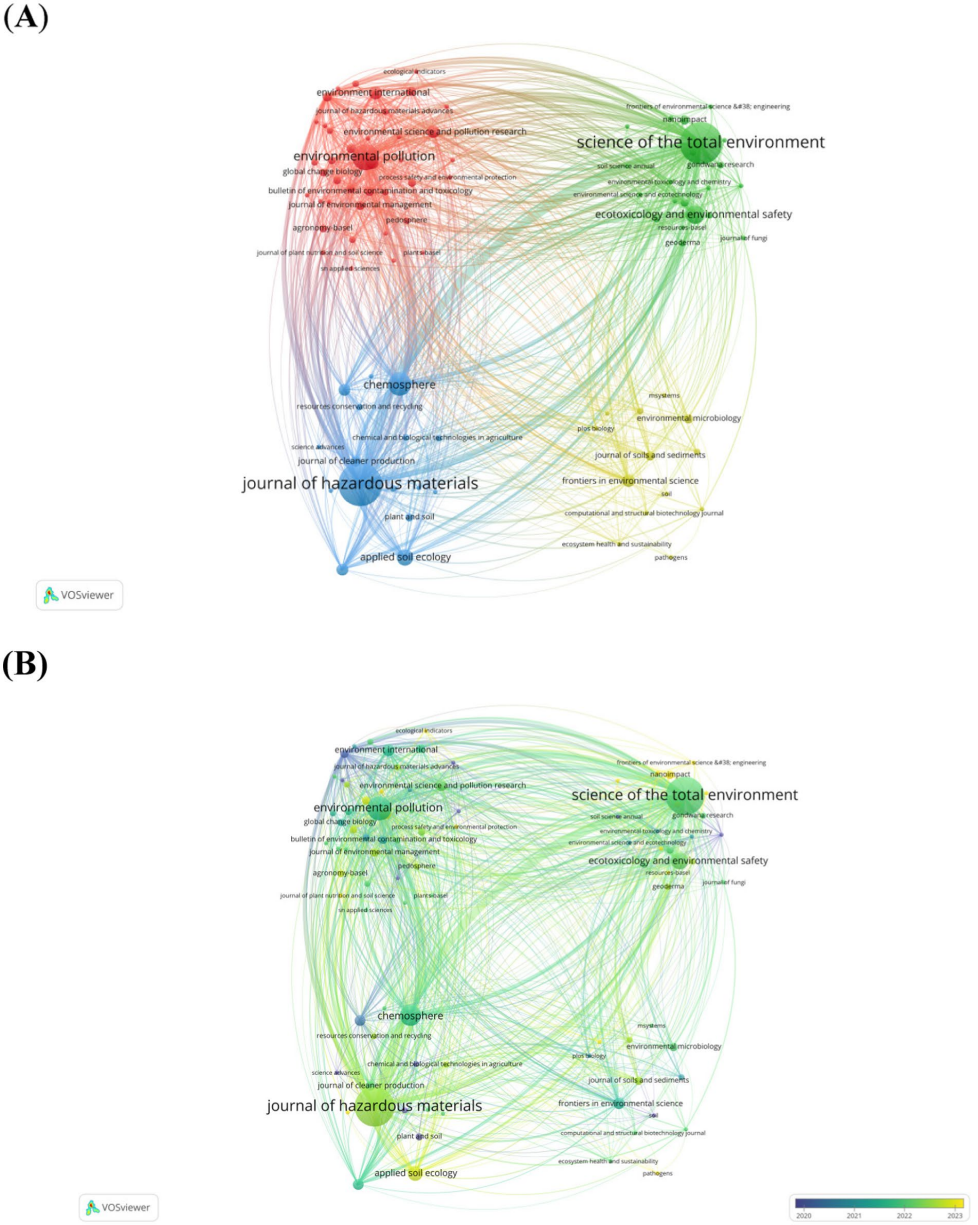

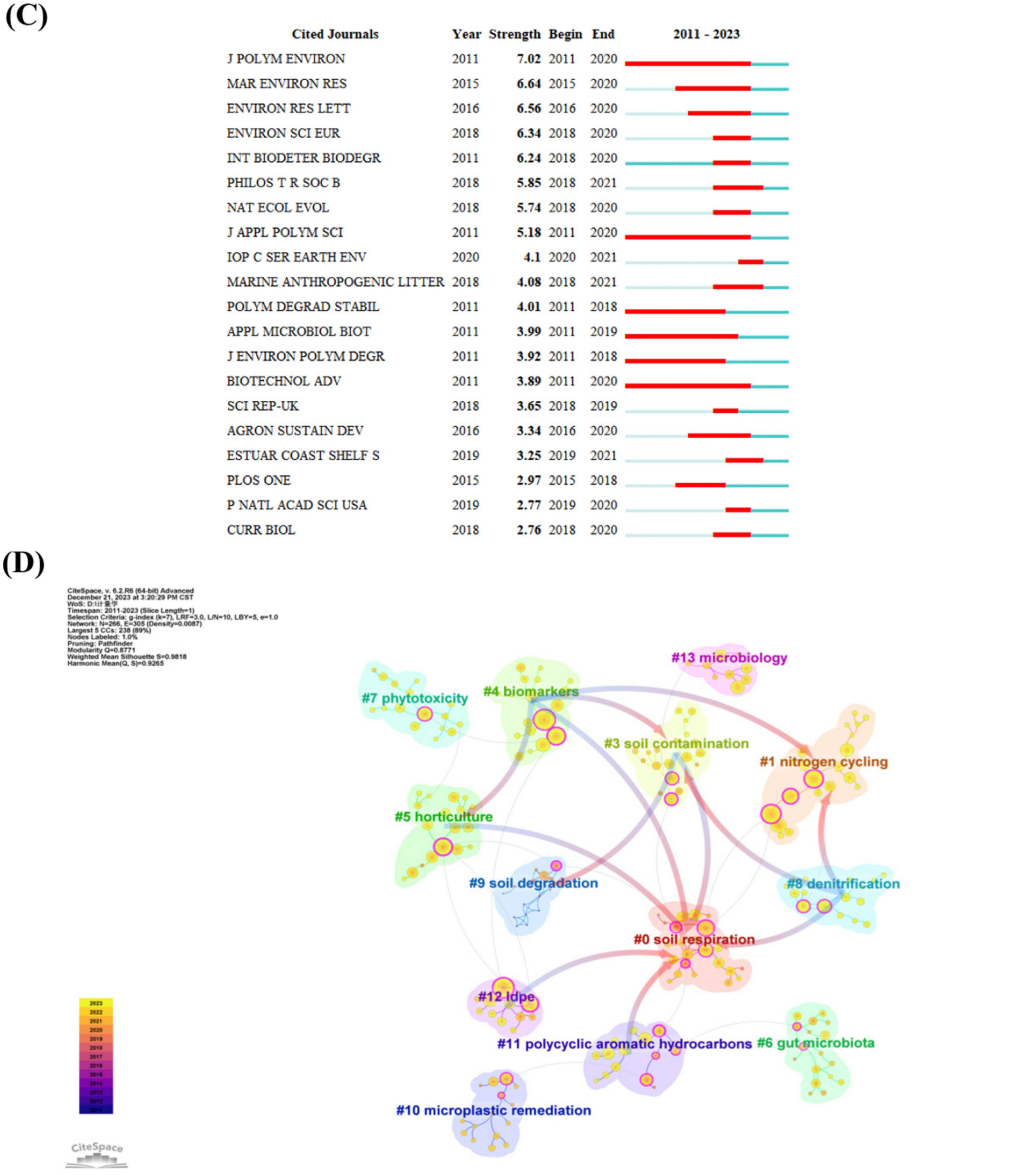
**(A)** timeline of journal publications **(B)**, the top 20 cited journals with the strongest burst citations **(C)**, and citation clusters **(D)**. In the clustering and timeline view map, each circle and its corresponding label constitute a node. The size of the circle corresponds to the frequency of the keywords, the color of the sphere indicates the average year of publication, as indicated by the color gradient in the lower right corner. Blue signifies journals with earlier publications, with *Advances and International Biodeterioration & Biodegradation* showing an early average publication time. Conversely, yellow denotes journals with more recent publications, with *Critical Reviews in Environmental Science and Technology*, *Ecological Indicators*, and *Environmental Engineering Research* representing relatively new entries in the field. In the citation clustering diagram, each color block represents a cluster group, with the cluster number being inversely proportional to the cluster size.

During 2011–2023, the leading journal that published the most articles was *Journal of Hazardous Materials*, followed by *Science of the Total Environment* and *Environmental Pollution,* with a total of 76 and 30 publications, respectively (Table 3; Figure 4A). In addition, the *Chemosphere*, *Ecotoxicology and Environmental Safety*, *Applied Soil Ecology*, *Environment International*, *Environmental Research*, *Environmental Science and Pollution Research*, and *Frontiers in Environmental Science* were also important sources. Further analysis indicated that there was a notable shift in journals during the examined period. The earlier studies mainly published on *Science Advances* and *International Biodeterioration & Biodegradation*, whereas the latest studies were published on *Critical Reviews in Environmental Science and Technology*, *Ecological Indicators*, and *Environmental Engineering Research* (Figure 4B). Burst analysis revealed that *Journal of Polymers and the Environment* experienced the strongest emergence, followed by *Marine Environmental Research*, with a bursting strength of 6.64 (Figure 4C).

**Table 3:** Top 25 influential journals with the number of published papers, Impact factor, and the position of the journal in its category.

Rank	Journal	TP	Links	TLS	IF	Quartile
1	J Hazard Mater	80	84	1,587	13.6	Q1
2	Sci Total Environ	76	87	1,582	9.8	Q1
3	Environ Pollut	30	78	905	8.9	Q1
4	Chemosphere	23	67	523	8.8	Q1
5	Ecotox Environ Safe	15	46	256	6.8	Q1
6	Appl Soil Ecol	12	48	252	4.8	Q2
7	Environ Int	8	52	234	11.8	Q1
8	Environ Res	8	30	137	8.3	Q1
9	Environ Sci Pollut R	7	30	136	5.8	Q1
10	Front Env Sci-Switz	7	54	177	4.6	Q2
11	Front Microbiol	6	51	164	5.2	Q2
12	Soil Biol Biochem	6	57	295	9.7	Q1
13	J Clean Prod	5	33	122	11.1	Q1
14	B Environ Contam Tox	4	45	115	2.7	Q3
15	Environ Microbiol	4	21	46	5.1	Q2
16	Environ Sci Technol	4	80	468	11.4	Q1
17	J Environ Manage	4	35	101	8.7	Q1
18	J Soil Sediment	4	29	95	3.6	Q2
19	NanoImpact	4	22	55	4.9	Q2
20	Sustainability-Basel	4	30	86	3.9	Q2
21	TrAC-Trend Anal Chem	4	47	139	13.1	Q1
22	Agronomy-Basel	3	21	40	3.7	Q1
23	Curr Opin Environ Sci Health	3	27	47	8.1	NA
24	Environ Technol Inno	3	22	66	7.1	Q1
25	Front Plant Sci	3	35	89	5.6	Q1

TP, total number of publications; TLS, total link strength; IF, impact factor; NA, the position of this journal has not been confirmed.

### 3.5 Highly cited references and co-cited references

**Figure 5:**
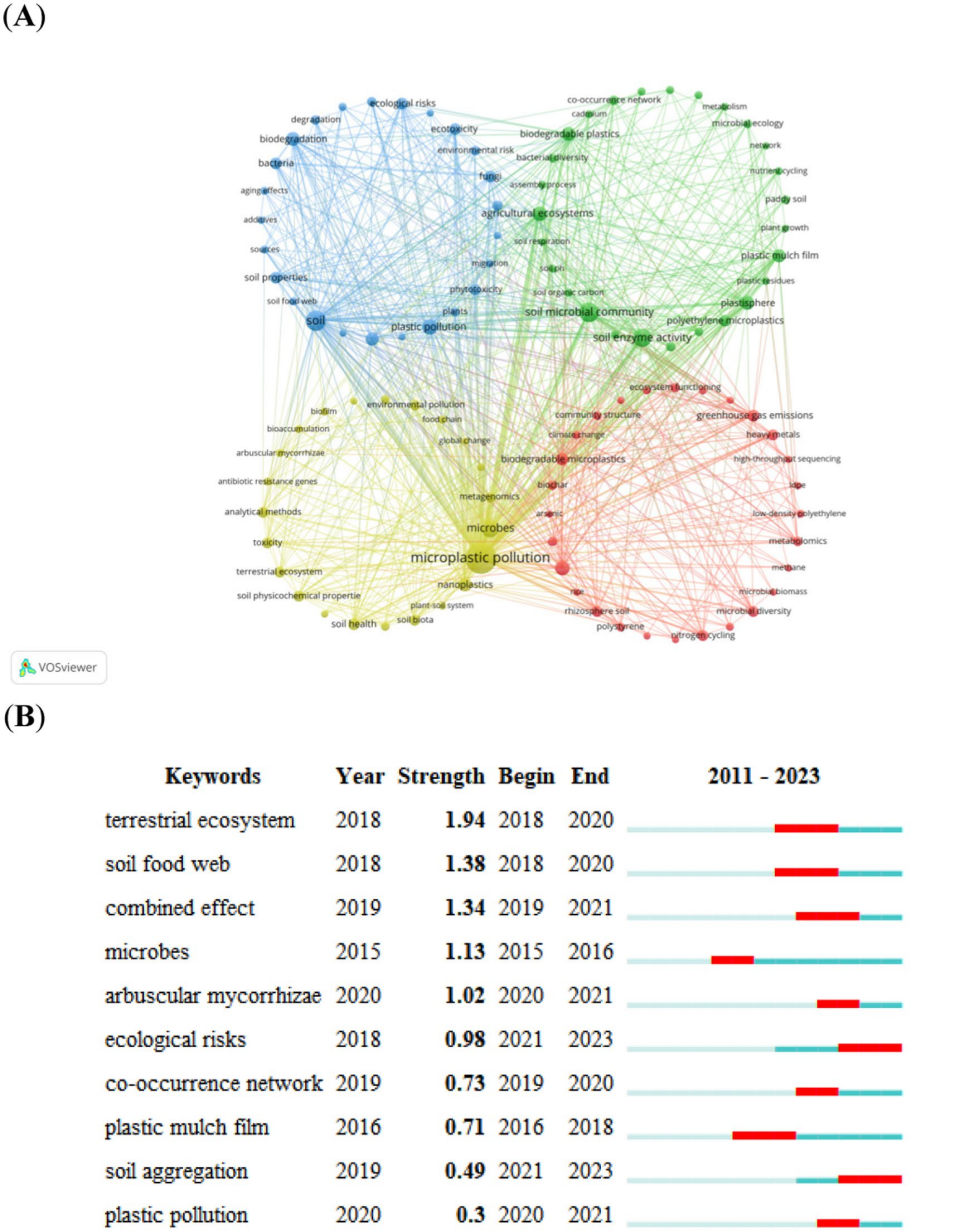
Co-occurrence keyword cluster network visualization of the author keywords using VOSviewer software **(A)**, and the top ten keywords with the strongest citation bursts maps using CiteSpace **(B)**. The minimum co-occurrence frequency of keywords was 3. To be clearer, only the most abundant keywords were shown in the co-occurrence network. Specifically, out of 1,110 keywords (1,004 after removing duplicates), a total of 103 keywords were selected to create a visual map. Each circle in the map represents a node, with the circle’s size indicating the frequency of that keyword. The thickness of the circle’s line represents the strength of the relationship between the keywords. The nodes are color-coded based on different research directions, determined using a clustering algorithm that calculates the similarity between keywords. Similar keywords are grouped together, and the specific cluster names can be summarized separately. The colors of each cluster describe each topic. The red cluster shows the highest interest in soil bacterial community, while the yellow cluster focuses on micro-plastic pollution. The green cluster highlights research on the soil microbial community, while the blue cluster emphasizes soil-related studies. In the top ten keywords with the strongest citation bursts, the blue line represents a time interval, while the red line highlights periods in which a particular keyword experienced a burst of activity or increased attention. The starting year and duration reflect the starting time and duration of the keywords that attracted widespread attention.

The co-cited publications were clustered into 14 categories (Figure 4D), namely #0 soil respiration, #1 nitrogen cycling, #2 plastic degradation, #3 soil contamination, #4 biomarkers, #5 horticulture, #6 gut microbiota, #7 phytotoxicity, #8 denitrification, #9 soil degradation, #10 micro-plastic remediation, #11 polycyclic aromatic hydrocarbons, #12 LDPE, and #13 microbiology. The most cited paper was a research article titled “Microplastics can change soil properties and affect plant performance,” which has been cited 205 times until January 1, 2024, based on the WoS database (Table 4). This study investigated the effects of six different microplastics (polyester fibers, polyamide beads, and four fragment types: polyethylene, polyester terephthalate, polypropylene, and polystyrene frag) on a broad suite of proxies for soil health and performance of spring onion (*Allium fistulosum*), and suggested that the pervasive micro-plastic contamination in soil May impact plant performance and further threat agroecosystems and terrestrial biodiversity As illustrated in the Figures 5A,B, the cluster-based co-citation network was found to be a well-structured and a sufficiently credible network (*Q* = 0.8771, *S* = 0.9818).

**Table 4:** Top 20 most cited papers.

Rank	Citation	Degree	Centrality	Sigma	First Author	Year	Source	DOI
1	205	5	0.15	1	Machado AAD	2019	Environ Sci Technol	10.1021/acs.est.9b01339
2	197	5	0.3	2.88	Machado AAD	2018	Environ Sci Technol	10.1021/acs.est.8b02212
3	172	3	0.36	1	Huang Y	2019	Environ Pollut	10.1016/j.envpol.2019.112983
4	154	6	0.33	1	Fei YF	2020	Sci Total Environ	10.1016/j.scitotenv.2019.135634
5	139	4	0.29	1	Boots B	2019	Environ Sci Technol	10.1021/acs.est.9b03304
6	135	6	0.37	3.29	Machado AAD	2018	Global Change Biol	10.1111/gcb.14020
7	134	6	0.58	1	Qi YL	2018	Sci Total Environ	10.1016/j.scitotenv.2018.07.229
8	123	4	0.38	74.95	Liu HF	2017	Chemosphere	10.1016/j.chemosphere.2017.07.064
9	116	3	0.02	1	Zhang MJ	2019	Sci Total Environ	10.1016/j.scitotenv.2019.06.108
10	114	2	0.32	1	Ren XW	2020	Environ Pollut	10.1016/j.envpol.2019.113347
11	114	4	0.02	1.18	Zhang GS	2018	Sci Total Environ	10.1016/j.scitotenv.2018.06.004
12	100	2	0.02	1	Qi YL	2020	J Hazard Mater	10.1016/j.jhazmat.2019.121711
13	97	7	0.11	1.69	Bläsing M	2018	Sci Total Environ	10.1016/j.scitotenv.2017.08.086
14	94	2	0.06	1	Seeley ME	2020	Nat Commun	10.1038/s41467-020-16235-3
15	93	1	0	1	Huang Y	2020	Environ Pollut	10.1016/j.envpol.2020.114096
16	92	3	0.09	1	Chen HP	2020	Chemosphere	10.1016/j.chemosphere.2019.125271
17	91	5	0.12	1	Wang FY	2020	Chemosphere	10.1016/j.chemosphere.2020.126791
18	86	3	0.1	1	Wang FY	2022	J Hazard Mater	10.1016/j.jhazmat.2021.127531
19	85	2	0.01	1	Guo JJ	2020	Environ Int	10.1016/j.envint.2019.105263
20	84	2	0.01	1.05	Ng EL	2018	Sci Total Environ	10.1016/j.scitotenv.2018.01.341

### 3.6 Keywords

**Figure 6:**
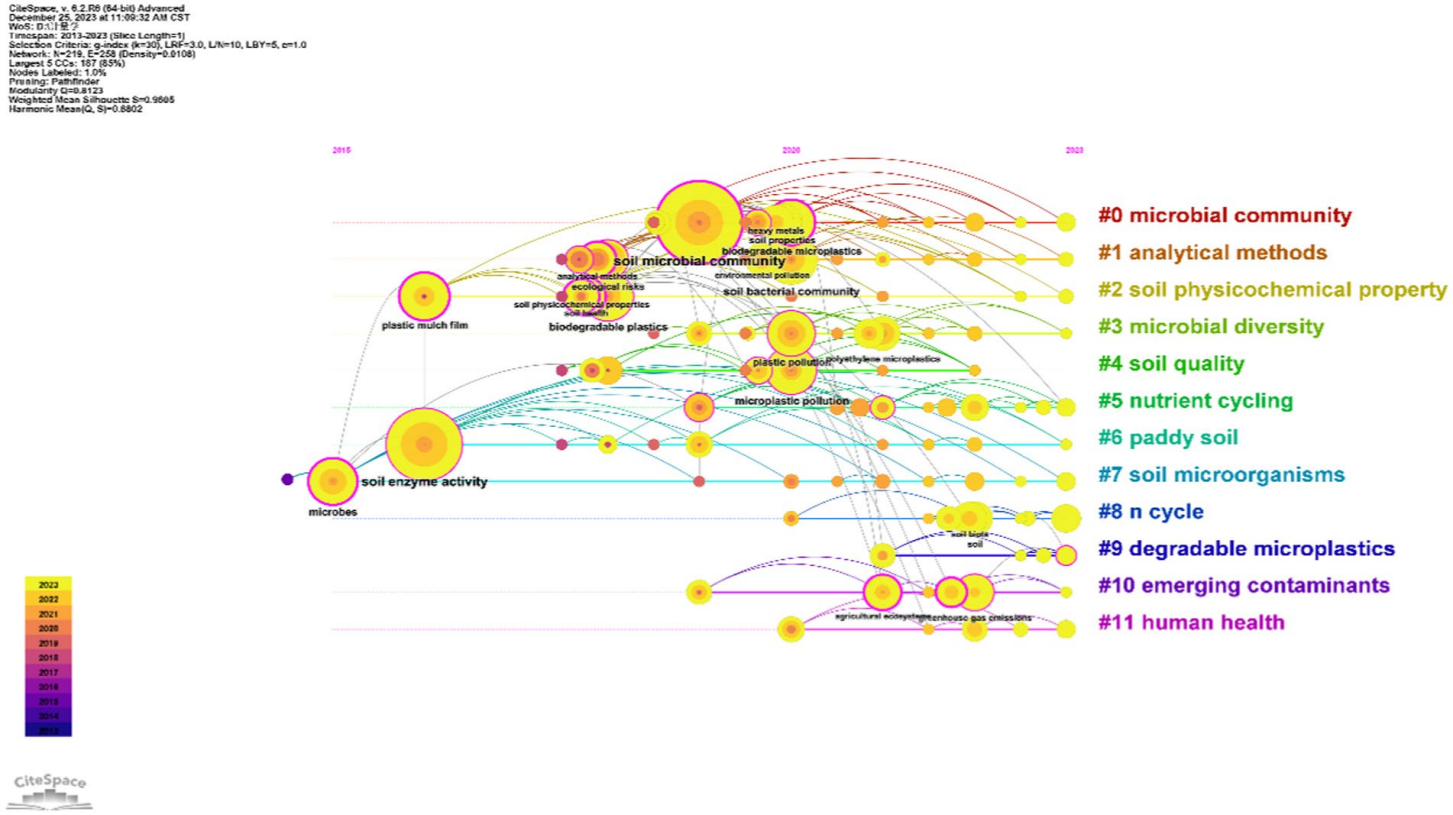
The timeline review of keywords. The size of the superimposed balls, indicating the corresponding ball on the annual ring line, is directly proportional to the frequency of keywords. The connection between keywords represents their co-occurrence. In the visualization, purple represents keywords that appeared earlier, while yellow represents those that appeared recently. The overlay color represents the keywords that appeared in a specific year. Within the network, rose-red nodes hold significant centrality as they occupy central positions and act as hubs. Keywords belonging to the same cluster are positioned along the same horizontal line. The occurrence time of each keyword is displayed at the top of the view, with more recent times towards the right. This graphical representation allows us to determine the number of keywords in each cluster, indicating the importance of the clustering field. Additionally, it provides insights into the time span of keywords within each category.

A total of 1,110 keywords were identified, and 1,004 keywords were obtained after excluding duplications and words not-related to the target terms of the present study, as well as merging synonymous keywords. Consequently, 103 keywords reached the minimum limit of three occurrences and exposed to final analysis. The top ten frequently occurring keywords were “soil bacterial community,” “biodegradable micro-plastics,” “green gas emissions,” “heavy metals,” “rhizosphere soil,” “microbial diversity,” “nitrogen cycling,” “ecosystem functioning,” “metabolomics,” and “soil quality.” These ten words also have the most connections with the other keywords in the dataset. The clustering analysis of keywords indicate that studies regarding interactions between soil microbial community and plastic contaminant are divided into four major research themes (Figure 6A; Supplementary Table S2). The first cluster (red) contains 28 author keywords: soil bacterial community, biodegradable micro-plastics, greenhouse gas emissions, heavy metals, rhizosphere soil, microbial diversity, nitrogen cycling, ecosystem functioning, metabolomics, soil quality, etc. The second cluster (yellow) contains 27 author keywords: soil microbial community, soil enzyme activity, agricultural ecosystems, plastisphere, biodegradable plastics, plastic mulch film, polyethylene micro-plastics, soil aggregation, co-occurrence network, bacterial diversity, etc. The third cluster (green) contains 25 author keywords: soil, plastic pollution, biodegradation, polyethylene, ecological risks, ecotoxicity, fungi, soil property, bacteria, phytotoxicity, etc. The fourth cluster (blue) contains 23 authors’ keywords: micro-plastic pollution, microbes, nano-plastics, soil health, analytical methods, soil physicochemical properties, toxicity, soil biota, terrestrial ecosystem, environmental pollution, etc. Overall, soil bacterial community, micro-plastic pollution, soil microbial community, and soil was the hottest research topics in these four themes, respectively. The time-series keyword co-occurrence network showed that all examined keywords can be divided into 12 clusters, namely #0 microbial community, #1 analytical methods, #2 soil physicochemical property, #3 microbial diversity, #4 soil quality, #5 nutrient cycling, #6 paddy soil, #7 soil microorganisms, #8 nitrogen cycle, #9 degradable micro-plastics, #10 emerging contaminants, and #11 human health. The cluster #0 is microbial community, and its publications are between 2017 and 2022. The “soil microbial community,” “biodegradable micro-plastics,” “soil properties,” “heavy metals,” etc. are included in this cluster. The cluster #1 is analytical methods, and its publications are between 2018 and 2023. This cluster contains 21 words, such as “ecological risks,” “terrestrial ecosystem.” The cluster #2 is soil physicochemical properties. The burst analysis of keywords displayed that “ecological risks” and “soil aggregation” are the keywords with strongest burst during the last five years (Figure 5B). Keywords, such as “ecological risks” and “soil aggregation” have showed burst increase during 2021–2023 (Figure 5B). The cluster #3 is microbial diversity. The cluster #4 is soil quality. The cluster #5 is nutrient cycling. The cluster #6 is paddy soil. The cluster 7 is soil microorganisms. The cluster#8 is N cycle. The cluster #9 is degradable microplastics. The cluster #10 is emerging contaminants, and the cluster #11 is human health. Overall, the studies on soil, plastic pollution, biodegradation, and soil microbial communities are a hot topic in the field of environmental science. The analysis of keyword co-occurrence networks shows that these topics are interconnected and have been the focus of research in recent years. The emergence of keywords related to degradable micro-plastics, emerging contaminants, and human health also indicates a growing interest in understanding the impacts of pollution on the environment and human well-being. This analysis provides valuable insights into the current trends and challenges in environmental science and highlights the need for continued research and innovation in this field (see Figure 6).

Thematic evolution analysis showed that the studying focus varied greatly during the examined periods (Supplementary Figure S2). The newly interests are the ecological risks and mechanisms of bio-gradable plastic residues on soil microbial community and associated functions in agroecosystem, the combined effects of plastic residues and other emerging pollution on enzymatic activity, soil microbial diversity in plastisphere and rhizosphere, phytotoxicity, soil food webs and soil health as well as the potential effects of biochar on mitigation of plastic pollution (Supplementary Figures S2B–E).

## 4 Discussion

To provide a better understanding of the current conditions and reveal future research directions of the impacts of plastic pollution on soil microbial communities, a bibliometric analysis of the publishing trends, including the countries, institutional collaborations, author collaborations, keywords, and hotspots was performed.

### 4.1 Implications of performance analysis

The observed steady increase in publications particularly from 2018 to 2023 (Figure 1) and from growing numbers of countries and institutions indicates that the impacts of plastic contamination on soil microorganisms have attracted widespread attention from scholars and have become a globally involved research topic. A likely reason for the tremendous increase in the publications is that the calling for due attention on ecological risks of microplastics in 2016 (Rochman et al., 2016) has received positive response. As evidenced by the findings, China was the most influential and productive country with the highest number of publications and related research institutions (Table 2). This result implies that China has attached a great importance to plastic pollution, and has become the center in the field of ecological impacts of plastic contaminant on soil microorganisms. The total citations and publications of a research institution reflect, to a certain extent, the studying scale and capacity in the field, as well as the close degree of cooperation between domestic and foreign research institutions (Zhang T. et al., 2023). Our findings suggested that the studying on the impacts of plastic pollution on soil microbial communities is an interdisciplinary field and formed relatively stable research center.

Recognizing collaborative networks in research across various countries, institutions, and authors is crucial (Zhou et al., 2018) as it aids in identifying the foremost and most innovative countries, institutions, and authors in this domain (Yu and Chen, 2021). Networking among countries and institutions helps to provide directions for further research (Wahyuningrum et al., 2023). Furthermore, evaluating the mode and extent of collaboration can elucidate the collaborative relationships at different stages (Lin, 2024). The impact assessment and mitigation of plastic contaminants are believed to be a multifaceted and comprehensive research topic, necessitating wide collaboration for future research. Although cooperative relationships between some countries, such as between China and the United States, were observed, the breadth and intensity of cooperation between institutions are not ideal. Clearly, this situation will hinder the development of the research field in the long run. Enhancing academic collaboration and exchange with other nations leads to mutually beneficial outcomes (Geng et al., 2022). Although authors with similar backgrounds in terms of nationality and institution tend to collaborate more frequently and easily (Zhang T. et al., 2023), cross-background, cross-institutional, and cross-national collaboration should be prioritized. Interdisciplinary collaboration is particularly are beneficial for the mutual learning among different teams and contribute to the rapid advancement and diverse development of ecological risks assessment and mitigation of plastic contamination. Therefore, we strongly recommend that research institutions in various countries carry out extensive cooperation and communication to jointly promote the development of mitigation strategies.

The assessment of research impact and researcher interest in scientometric literature heavily relies on citation metrics (Bagdi et al., 2023). Traditional citation metrics often emphasize established researchers and prominent journals, potentially overlooking emerging scholars and interdisciplinary research. In contrast, altmetrics can highlight impactful work that resonates with the public, policymakers, and practitioners, thereby democratizing the recognition process. Moreover, altmetrics enable real-time tracking of research influence, providing immediate feedback to researchers regarding how their work is being received and utilized. This feedback can encourage more dynamic and responsive research practices, enabling scholars to adjust their focus and dissemination strategies based on ongoing engagement and feedback from a broader audience. Additionally, the adoption of altmetrics can catalyze the development of new tools and platforms for research dissemination and collaboration. By leveraging social media, academic networks, and digital repositories, researchers can enhance the visibility and accessibility of their work, fostering greater collaboration and innovation across disciplines. Furthermore, the integration of altmetrics into research assessment can promote greater transparency and inclusivity in recognizing scholarly contributions. By embracing a more diverse array of metrics, the academic community can better capture the multifaceted nature of scholarly influence and contribution, ultimately advancing the goals of knowledge dissemination and societal advancement.

### 4.2 Critical knowledge gaps

Identifying critical knowledge gaps in the studying of the impacts of plastic pollution on soil microorganisms is crucial for advancing our understanding and implementing effective strategies. Overall, the effects of plastic residues on soil microbes varied significantly across polymer type, particle shape, size, elemental composition and concentration, exposure duration, soil properties, land-use type and target object (Bucci et al., 2020; Qiu Y. et al., 2022; Sun et al., 2022; Li H. Q. et al., 2021; Liu et al., 2023; Liu M. et al., 2024; Jia et al., 2024). Microplastics increased soil microbial biomass, substantially reduced soil bacterial diversity (as measured by the Shannon index) and altered the microbial community composition, with their impact on soil bacteria being more pronounced than on soil fungi (Liu M. et al., 2024). Our analysis indicates that due to limited empirical data, the effects of micro(nano)plastics on soil microbes have not been fully characterized.

A critical knowledge gaps is that the findings from laboratory experiments need to be further validated under field conditions. Firstly, while numerous studies have documented the presence and physical effects of micro(nano)plastics in soil environments, their toxic effects on microbial health and function have not been fully explored. For instance, studies have shown that microplastics can alter soil physical properties, such as bulk density, soil aggregation stability and soil moisture (Zhang B. et al., 2020; Qiu Y. et al., 2022), soil pH (Song et al., 2024), soil organic carbon content (Zhao et al., 2021; Guo et al., 2022) as well as nutrient availability (Yang M. et al., 2021; Yang J. et al., 2021; Yu et al., 2020), potentially impacting microbial activity and diversity (Rillig et al., 2019b; Hanif et al., 2024). However, direct assessments of toxicity, such as changes in microbial cell viability, metabolic activity, and gene expression induced by micro(nano)plastic exposure, are still limited. Experimental studies on the biochemical and physiological responses of soil microbes to micro(nano)plastics are essential. For instance, it is crucial to determine whether micro(nano)plastics induce oxidative stress, disrupt cell membranes, or interfere with essential metabolic processes in soil microbes (Zhu et al., 2018). Furthermore, most existing studies on the impacts of micro(nano)plastics on soil microbial community have used a certain type of virgin polymer. In contrast, under field conditions, soil microbial communities are often exposed to a mixture of different micro(nano)plastics, covering a range of degradation gradients. Therefore, it is necessary to investigate how the concentration, particle size, shape, and chemical composition of micro(nano)plastics affect their toxicity. Such studies can reveal the mechanisms underlying microbial stress induced by micro(nano)plastic and help determine which specific microbial groups are particularly susceptible to these pollutants (Qi Y. et al., 2020). In addition, most existing studies use concentrations higher than those found in real environmental conditions (Shen et al., 2019; Wang W. et al., 2021; Wang L. et al., 2021). Furthermore, most studies involve a single soil type, whereas the responses of soil microbial communities to micro(nano)plastics vary greatly under field conditions, influenced by the diversity of microbial taxa, the heterogeneity of soil types, and intensive anthropogenic activities. It is well known that different soil types exhibit distinct differences in physicochemical properties, microbial diversity, and functional gene abundance (Li H. Z. et al., 2021; Dong et al., 2024a). Soil microbial communities exhibit selective response to plastic residues. Different microbial species and functional groups show varying degrees of sensitivity to micro(nano)plastic pollution. For instance, some bacteria May possess metabolic pathways that enable them to degrade certain types of microplastics, whereas others May be inhibited or killed by the same contaminants (Rochman et al., 2016). This variability in microbial responses makes it more complex to generalize the ecological impacts of micro(nano)plastics in different soil ecosystems. Moreover, soil characteristics, such as texture, soil water content, organic matter content and pH, can modulate the effects of micro(nano)plastics on microbial communities. For instance, soils with high organic matter content May adsorb micro(nano)plastics, reducing their bioavailability and toxicity to microbes (Salam et al., 2023). Conversely, sandy soils with low organic content cannot buffer these impacts as effectively, leading to greater microbial stress. Understanding the interactions between soil properties and micro(nano)plastics is crucial for predicting the ecological outcomes of microplastic pollution in different environmental contexts. Given this complexity, comprehensive studies involving a wide range of microbial taxa and different soil types are needed. Such studies should aim to identify patterns and drivers of microbial response to micro(nano)plastics, helping elucidate factors determining susceptibility and resilience. By incorporating microbiological, soil science, and environmental chemistry approaches, future studies can comprehensively understand the ecotoxicological effects of micro(nano)plastics on soil ecosystems (de Souza Machado et al., 2018a).

The long-term ecological impacts of microplastics on soil microbial communities are another critical knowledge gap. Most existing studies concentrate on short-term effects. Short-term laboratory experiments, while valuable for initial insights, cannot reflect the dynamic interactions and cumulative effects that occur over a long period in natural environments. For instance, a recent study reported that short-term exposure to pollutants significantly affected soil bacterial functions, while long-term exposure did not exert significant impact on soil bacterial functions (Wu Z. et al., 2024). In contrast, long-term studies under field condition are essential to capture seasonal variations, microbial succession, and the gradual accumulation of microplastics in soils, providing a more comprehensive picture of their ecological impacts (Liu et al., 2023). Plastic accumulation and persistence in soil can last for many decades (Temporiti et al., 2022). Short-term studies hinder our ability to generalize findings and assess the chronic effects of microplastic pollution on soil health and functionality (Rochman et al., 2016). Over time, the microplastics gradually fragment and degrade into smaller particles, including nanoplastics, which can alter their toxicity and bioavailability (Ya et al., 2021; Wu J. Y. et al., 2024). Long-term field studies can reveal these processes, showing how the aging and weathering of microplastics affect their interactions with soil microbes. Furthermore, such studies can help determine the adaptability or resistance potential of microbes to micro(nano)plastic pollution, enhancing a deeper understanding of ecosystem resilience. The complexity and variability of soil ecosystems also require long-term monitoring and field studies to understand the comprehensive impact of micro(nano)plastics on soil microbial communities. By evaluating the long-term and synergistic impacts of micro(nano)plastics, scholars can better predict their ecological consequences and provide information for risk assessments and remediation efforts. This knowledge is crucial for developing sustainable agricultural practices and environmental management strategies, protecting soil health and ensuring the continued provision of important ecosystem services.

In addition, it is noteworthy noting that microplastics and/or nanoplastics can absorb other pollutants, including heavy metals. Microplastics rarely exist alone; they interact with heavy metals, pesticides (Fu et al., 2024; Wu C. et al., 2024), and organic pollutants (Ya et al., 2023), which May exacerbate their toxic effects on soil microbial communities. Therefore, it is a necessity to explore the potential interactions between microplastics and other soil pollutants, such as heavy metals (Meng et al., 2023; Li et al., 2024; Song et al., 2024; Sun et al., 2024) and pesticides (Fu et al., 2024), which May enhance or diminish their ecological impacts on soil ecosystem. Besides, the toxicity caused by plastic additives as well as the indirect effects on soil properties, such as soil water content (Qiu Y. et al., 2022) and soil organic carbon (Zhao et al., 2021; Guo et al., 2022; Salam et al., 2023), and plant performance (Qi et al., 2018; Jiang et al., 2019; Zhang L. et al., 2022) during plastic degradation, would also have a significant impact on soil microbial community (Boots et al., 2019; de Souza Machado et al., 2019). To deepen our understanding of the specific effects and underlying mechanisms, of plastic debris on soil microbes and/or microbial communities. It is necessary to include more types of polymers, shapes, particle sizes, degradation stages and concentrations of plastic debris into consideration. For instance, nanoplastics, May have unique impacts that require further investigation. Meanwhile, despite microplastics being recognized as a pervasive pollutant in soil ecosystems, our understanding of their specific interactions with soil microbial communities at a molecular and cellular level is still relatively unclear. Current studies mainly focus on the presence and quantification of microplastics in soils, which leaves a significant knowledge gap in understanding how these particles influence microbial physiology and metabolism. For instance, although it is known that microplastics can serve as vectors for chemical pollutants and pathogens, the exact mechanisms of interaction between these pollutants and microbial cell membranes, intracellular processes, and genetic material are poorly understood (Rillig et al., 2019b). Advanced molecular techniques, such as metagenomics, proteomics, and metabolomics, are urgently needed to dissect these complex interactions. These approaches can reveal changes in microbial gene expression, shifts in metabolic pathways, and changes in community composition induced by microplastic exposure (Tiwari et al., 2022). Furthermore, studying the physical interactions between microplastics and microbial cells, such as adhesion, biofilm formation, and particle ingestion, is crucial for understanding the broader ecological implications (Qiu X. et al., 2022). Without a detailed understanding from molecular and cellular perspectives, our ability to predict the ecological consequences of micro(nano)plastics in soils will be severely limited. Besides, a comprehensive understanding the ecological risks of micro(nano)plastics, particularly for nonbiodegradable nanoplastics the perspective of soil food web is also needed (Wei et al., 2022). Exploring the potential impacts of nanoplastics on soil microorganisms and their roles in soil nutrient cycling could provide valuable insights into the overall ecological consequences of nanoplastic pollution in terrestrial ecosystems. Studying the interactions between nanoplastics and soil biota at the molecular level May reveal novel mechanisms underlying the persistence and bioaccumulation of nanoplastics in soil environments. This deeper understanding is crucial for developing effective mitigation strategies to safeguard soil health and ecosystem resilience. Finally, soil, soil microbes and plants are closely related and interact constantly, and thus it is advisable to unravel the general response patterns of ecosystems to plastic pollution in the view of plant–soil-microbial systems (Jia et al., 2024).

The fourth critical knowledge gap is to better understand how changes in microbial communities resulted from plastic contaminants affect soil ecosystems and their functions. Due to the complex interactions between plastic residues, soil and soil microorganisms, and the mechanism of the effect of micro(nano)plastics on soil microorganisms is not clear at present (Zhou Y. et al., 2020; Qi et al., 2024). Plastic pollution is expected to induce shifts in soil microbial community composition and its mediated ecological processes through pH (Song et al., 2024; Zhang et al., 2024), soil moisture (Qiu Y. et al., 2022), soil organic matter content (Zhao et al., 2021; Salam et al., 2023; Iqbal et al., 2024a). The shifts in community structure can further change microbial function (Huang et al., 2019; Fei et al., 2020; Li H. Z. et al., 2021; Dong et al., 2024a; Dong et al., 2024b; Liu et al., 2024a), microbial interactions (Hu et al., 2022; Shi et al., 2022a; Zhou et al., 2024) as well as the complex interplay of microbial processes in the soil environment (Zhang et al., 2024). It is still unclear how changes in microbial communities caused by plastic contaminants affect soil ecosystems and their functions. Therefore, investigating the influence of plastic residues on essential soil processes like nutrient cycling is crucial for assessing broader ecological consequences (Shi et al., 2022b; Zhu D. et al., 2022; Zhu F. et al., 2022; Liu et al., 2024a). Furthermore, recent studies have examined the effects of micro(nano)plastics on greenhouse gasses (Gao et al., 2021; Zhang et al., 2022; Rauscher et al., 2023; Iqbal et al., 2024b; Su et al., 2024; Sunil et al., 2024) and climate change (Shen et al., 2020), whereas the causal relationship between micro(nano)plastics and climate change needs further exploration (Chia et al., 2023). Finally, studying the spread of plastic-associated contaminants through the food web is essential for understanding the broader ecological impacts of soil plastic pollution. Studies have shown that plastic pollution significantly affects plant health (Khalid et al., 2020; Guo et al., 2024; Qi et al., 2018) and higher trophic levels (Gao et al., 2024; Guo et al., 2024; Kwak et al., 2024), whereas complex interactions exist between plants, soil microorganisms and animals need further investigation. Investigating the combined pollution of micro(nano) plastics and other contaminants on soil microbial functional groups and functional genes is currently a key research focus (Ya et al., 2021), with potential to advance the field in the coming years.

Furthermore, there are few studies addressing the interactions between micro(nano)plastics and soil microbial communities under environmental stresses, such as drought (Liu et al., 2024b; Lozano et al., 2021a; Lozano et al., 2024), elevated CO_2_ (Xu et al., 2021; Xu et al., 2023), and nitrogen deposition (Zhang S. et al., 2023). Therefore, investigating the effects of these environmental stressors on the interactions between micro(nano)plastics and soil microbial communities is crucial for understanding the potential risks associated with plastic pollution in terrestrial ecosystems. Investigating how drought conditions affect the behavior and fate of micro(nano)plastics in soil, as well as their effects on microbial community structure and function, can provide valuable insights into the resilience of soil ecosystems in the face of climate change challenges. Moreover, exploring the combined effects of elevated CO_2_ and nitrogen deposition on the interactions between micro(nano)plastics and soil microbial communities can provide a comprehensive understanding of how multiple stressors May exacerbate the ecological consequences of plastic contamination in soil environments. By unraveling the intricate relationships between environmental stressors and micro(nano)plastics in soil systems, we can better assess the long-term impacts on soil health, plant growth, and ecosystem function. In conclusion, integrating studies on the interactions between micro(nano)plastics and soil microbial communities under various environmental stressors are essential for advancing our knowledge of the ecological impacts of plastic pollution in terrestrial ecosystems. Future research efforts should focus on elucidating the mechanisms of these interactions, identifying potential mitigation strategies, and developing sustainable management practices to protect soil biodiversity and ecosystem resilience in the face of global environmental changes.

Finally, identifying soil microbes with potential to degrade microplastic and/or nanoplastics and developing sustainable alternatives to conventional plastics that reduce negative impacts on soil microorganisms, is also a critical knowledge gap. This requires interdisciplinary collaborations between microbiologists, environmental scientists, and material engineers. By harnessing the power of biotechnology and nanotechnology, innovative solutions can be developed for the degradation of microplastics and nanoplastics in soil ecosystems. Understanding the molecular interactions between soil microbes and plastic pollutants is crucial for designing customized biodegradation strategies. Furthermore, the development of environmentally friendly bioplastics derived from renewable resources can provide sustainable alternatives to traditional plastics, reducing the accumulation of harmful residues in the soil environments. In addition to technological advancements, educational initiatives and public awareness campaigns play a pivotal role in promoting responsible plastic use and waste management practices.

### 4.3 Hotspots and frontiers

Keywords, the most significant phrases in a particular field of study (Ye et al., 2020), serve as crucial markers for understanding patterns and trends in research that aid in comprehending shifts in focal points and development trends over time (Bagdi et al., 2023). Examining the joint presence of keywords is an effective approach to pinpointing research focal points in a specific field of study (Gao et al., 2020; Ranjbari et al., 2021). Moreover, performing cluster analyses on keyword is advantageous for attaining a comprehensive insight into past and present research concerns and identifying future research prospects (Wahyuningrum et al., 2023). The cluster analysis of the most frequently associated keywords spotlights soil bacterial communities, micro-plastic pollution, soil microbial communities, and soil as the most prevalent research subjects. Additionally, conducting a burst analysis on key terms aids in mapping out the progression of research highlights over time, and anticipating forthcoming research directions (Chen et al., 2014). The burst analysis of keywords supports that “ecological risks” and “soil aggregation” are the latest and most important studying topics of the impacts of plastic pollution on soil microorganisms.

The ecological risks of plastic pollution on soil microbial community and associated ecosystem functioning are a complex issue. To date the underlying mechanisms responsible for the impacts of microplastics on soil microbial communities has been relatively less explored (Huang et al., 2024), and thus further research is needed. Understanding the ecological risks of plastic pollution on soil microbial communities and associated ecosystem function, studies with unambiguous objectives, scientifically rigorous experiment design, appropriate sampling and analyzing techniques, as well as statistical method are needed. Meanwhile, a multidisciplinary approach combining ecological, microbiological, and environmental science is required.

Soil aggregation refers to the process by which soil particles bind together to form stable aggregates. These aggregates provide structural stability to the soil, improve water infiltration and retention, and create specific habitats for soil microorganisms. Plastics pollution can negatively impact soil aggregation by physically interfering with the formation and stability of soil aggregates (Qiu Y. et al., 2022). Plastics fragments can also hinder nutrient and water movement through the soil, affecting the availability of essential resources for soil microorganisms. Consequently, the disruption of soil aggregation due to plastic pollution can reduce the habitat quality for soil microbes, impair their functioning, and ultimately affect the overall soil health. Microplastic can affect soil organic matter, soil microbial community, enzymatic activities, and soil aggregation (Blöcker et al., 2020; Xiao et al., 2022). However, the impacts of plastic pollution on soil aggregation are still a controversial issue. Some studies found soil aggregation was negatively affected by microplastics (de Souza Machado et al., 2018b; Lozano et al., 2021b; Qiu Y. et al., 2022; Zhang Y. et al., 2022), one study demonstrated no significant effect (Liang et al., 2021), whereas another study reported that soil aggregation was increased by 21.7% (Lozano et al., 2021a). A likely reason for the inconsistency of microplastic pollution on soil aggregation would be related to differences in the aggregate-fraction method and soil organic matter. Furthermore, the interactions between microplastic and microbes is mutual, and the mechanisms driving their interactions is unclear and deserve due attention (de Souza Machado et al., 2018b; Lozano et al., 2021b; Qiu Y. et al., 2022). Finally, soil pH and plants also regulate the responses of soil microbes to plastics (Liu et al., 2023). Therefore, it is advisable to evaluate ecological risks of micro(nano) plastics based on whole ecosystem functions rather than individual function (Jia et al., 2024).

The impacts of plastics pollution on soil microorganisms at a fine-scale are a relatively new area of research that is considered a frontier. Studying the specific effects of plastic pollution on ecological risks and soil aggregation is crucial for a comprehensive understanding of how plastics pollution affects soil microorganisms. This research can lead to the identification of biomarkers, prediction of long-term effects, and the development of effective strategies to mitigate the damage caused by plastics pollution. In summary, understanding the ecological risks and soil aggregation is vital in comprehending the impacts of plastics pollution on soil microorganisms. Addressing these critical areas is essential for maintaining soil health, ecosystem functioning, and ensuring the sustainability of our planet.

### 4.4 Merits and limitations

This study has several unique merits. Firstly, studies addressing impacts of micro-plastics on soil microbial community were systematically analyzed using bibliometric for the first time to provide comprehensive guidance for scholars in the related research. Additionally, multiple bibliometric tools were used simultaneously to overcome their individual limitations and thus provided detailed and convincing evidence. Finally, bibliometric analysis provides more complete and objective insight into the hotspots and frontiers than traditional reviews.

While bibliometric analysis provides valuable quantitative insights into research trends, productivity, and impact, it is important to acknowledge its limitations in capturing the full depth and context of research content. Bibliometric methods primarily focus on measurable data such as publication counts, citation metrics, and collaboration networks. However, they often fall short in providing a comprehensive understanding of the qualitative aspects of research, such as the nuances of scientific discourse, the context of discoveries, and the broader implications of research findings. Additionally, it cannot provide an in-depth topic content (Paul and Criado, 2020; Wahyuningrum et al., 2023).

## 5 Conclusion and outlook

### 5.1 Conclusion

The increasing number of publications highlights the growing interest in the potential impacts of plastic contamination on soil biodiversity and ecosystem functioning, particularly on soil microbial communities. However, studies on the ecological risks of plastic pollution on soil microbial communities are still limited, and the mechanisms underlying these impacts are not yet clear. The leading countries investigating the ecological risks of plastic pollution on soil microorganisms are China, Germany, and the United States. Nevertheless, there is a need to enhance cooperation and communication among different countries and institutions. Davey L. Jones and Geissen Violette are suggested as potential collaborators in this field. Journals such as *Journal of Hazardous Materials*, *Science of the Total Environment*, *Environmental Pollution*, *Chemosphere*, *Ecotoxicology and Environmental Safety*, *Applied Soil Ecology*, *Environment International*, *Environmental Research*, *Environmental Science and Pollution Research*, and *Frontiers in Environmental Science* have played significant roles in exploring the interactions between soil microorganisms and plastics, with the *Journal of Hazardous Materials* being particularly influential, although improvements in quality and influence are still needed. The most cited paper in this area is “Microplastics can change soil properties and affect plant performance”. The main keywords of studying on the impacts of plastic pollution on soil microbial communities are soil, plastic pollution, biodegradation, and soil microbial communities. The potential hotspots for future research in this field are the ecological risks of biodegradable plastic on soil microbial diversity and functions.

### 5.2 Future research directions and management recommendations

Since the generalizable pattern of responses of soil microbes to contaminant due to the present of plastic residues remain poorly understood, more long-term field studies including more polymer types, element composition, size and ages in widescale are needed. Furthermore, to evaluate ecological risks of micro(nano) plastics from the perspective of soil food web with advanced multi-omics approaches is necessary. Meanwhile, a detailed content analysis of key publications identified through bibliometric methods to understand the themes, arguments and innovations is also necessary. In-depth case studies of specific research groups, institutions, or projects are expected to uncover the social, economic, and institutional factors influencing research productivity and impact. Engaging with researchers, policymakers, and other stakeholders through interviews and surveys to identify emerging research areas and priorities that May not yet be reflected in bibliometric data. Integrating quantitative bibliometric analysis with qualitative data collection and analysis to provide a more comprehensive and actionable understanding of the scientific landscape, thereby informing more effective research funding and policy decisions. In conclusion, establishing robust monitoring protocols to assess the presence and abundance of plastics in various soil types, along with the corresponding changes in microbial communities, is crucial. Collaboration among ecologists, microbiologists, materials scientists, and policymakers is essential to mitigate the environmental consequences of plastic pollution and safeguard the health and resilience of soil ecosystems.

## Data Availability

The original contributions presented in the study are included in the article/Supplementary material, further inquiries can be directed to the corresponding author.

## Footnotes

^1^ https://www.webofscience.com/wos/woscc/basic-search

## References

[ref1] Abbasi S. Moore F. Keshavarzi B. Hopke P. K. Naidu R. Rahman M. M. . (2020). PET-microplastics as a vector for heavy metals in a simulated plant rhizosphere zone. Sci. Total Environ. 744:140984. doi: 10.1016/j.scitotenv.2020.140984 32707415

[ref2] Accinelli C. Abbas H. K. Shier W. T. Vicari A. Little N. S. Aloise M. R. . (2019). Degradation of microplastic seed film-coating fragments in soil. Chemosphere 226, 645–650. doi: 10.1016/j.chemosphere.2019.03.161, PMID: 30959449

[ref3] Allen S. Allen D. Phoenix V. R. Le Roux G. Durántez Jiménez P. Simonneau A. . (2019). Atmospheric transport and deposition of microplastics in a remote mountain catchment. Nat. Geosci. 12, 339–344. doi: 10.1038/s41561-019-0335-5

[ref4] Aria M. Cuccurullo C. (2017). Bibliometrix: An R-tool for comprehensive science mapping analysis. J Informetr 11, 959–975. doi: 10.1016/j.joi.2017.08.007

[ref5] Bagdi T. Ghosh S. Sarkar A. Hazra A. K. Balachandran S. Chaudhury S. (2023). Evaluation of research progress and trends on gender and renewable energy: a bibliometric analysis. J. Clean. Prod. 423:138654. doi: 10.1016/j.jclepro.2023.138654

[ref6] Bahram M. Hildebrand F. Forslund S. K. Anderson J. L. Soudzilovskaia N. A. Bodegom P. M. . (2018). Structure and function of the global topsoil microbiome. Nature 560, 233–237. doi: 10.1038/s41586-018-0386-6, PMID: 30069051

[ref7] Banerjee S. Schlaeppi K. van der Heijden M. G. A. (2018). Keystone taxa as drivers of microbiome structure and functioning. Nat. Rev. Microbiol. 16, 567–576. doi: 10.1038/s41579-018-0024-1, PMID: 29789680

[ref8] Bardgett R. D. van der Putten W. H. (2014). Belowground biodiversity and ecosystem functioning. Nature 515, 505–511. doi: 10.1038/nature13855 25428498

[ref9] Beloe C. J. Browne M. A. Johnston E. L. (2022). Plastic debris as a vector for bacterial disease: An interdisciplinary systematic review. Environ. Sci. Technol. 56, 2950–2958. doi: 10.1021/acs.est.1c05405 35129968

[ref10] Bergmann M. Mützel S. Primpke S. Tekman M. B. Trachsel J. Gerdts G. (2019). White and wonderful? Microplastics prevail in snow from the Alps to the Arctic. Sci. Adv. 5:eaax1157. doi: 10.1126/sciadv.aax1157, PMID: 31453336 PMC6693909

[ref11] Blöcker L. Watson C. Wichern F. (2020). Living in the plastic age-different short-term microbial response to microplastics addition to arable soils with contrasting soil organic matter content and farm management legacy. Environ. Pollut. 267:115468. doi: 10.1016/j.envpol.2020.115468, PMID: 32891047

[ref12] Boots B. Russell C. W. Green D. S. (2019). Effects of microplastics in soil ecosystems: above and below ground. Environ. Sci. Technol. 53, 11496–11506. doi: 10.1021/acs.est.9b03304, PMID: 31509704

[ref13] Börner K. (2011). Science of science (Sci^2^) tool. Commun. ACM 54, 60–69. doi: 10.1145/1897852.189787121984822

[ref14] Brahney J. Hallerud M. Heim E. Hahnenberger M. Sukumaran S. (2020). Plastic rain in protected areas of the United States. Science 368, 1257–1260. doi: 10.1126/science.aaz5819, PMID: 32527833

[ref15] Bucci K. Tulio M. Rochman C. M. (2020). What is known and unknown about the effects of plastic pollution: a meta-analysis and systematic review. Ecol. Appl. 30:e02044. doi: 10.1002/eap.2044 31758826

[ref16] Bullard J. E. Ockelford A. O'Brien P. McKenna Neuman C. (2021). Preferential transport of microplastics by wind. Atmos. Environ. 245:118038. doi: 10.1016/j.atmosenv.2020.118038

[ref17] Chen C. (2006). Cite space II: detecting and visualizing emerging trends and transient patterns in scientifc literature. J Am Soc Inf Sci Tec 57, 359–377. doi: 10.1002/asi.20317

[ref18] Chen C. Dubin R. Kim M. C. (2014). Emerging trends and new developments in regenerative medicine: a scientometric update (2000 – 2014). Expert Opin Biol Th 14, 1295–1317. doi: 10.1517/14712598.2014.920813, PMID: 25077605

[ref19] Chia R. W. Lee J. Y. Lee M. Lee G. S. Jeong C. D. (2023). Role of soil microplastic pollution in climate change. Sci. Total Environ. 887:164112. doi: 10.1016/j.scitotenv.2023.164112, PMID: 37172846

[ref20] Cobo M. J. López-Herrera A. G. Herrera-Viedma E. Herrera F. (2012). Sci MAT: a new science mapping analysis software tool. J Am Soc Inf Sci Tec 63, 1609–1630. doi: 10.1002/asi.22688

[ref21] Corradini F. Meza P. Eguiluz R. Casado F. Huerta-Lwanga E. Geissen V. (2019). Evidence of microplastic accumulation in agricultural soils from sewage sludge disposal. Sci. Total Environ. 671, 411–420. doi: 10.1016/j.scitotenv.2019.03.368, PMID: 30933797

[ref22] de Souza Machado A. A. Kloas W. Zarfl C. Hempel S. Rillig M. C. (2018a). Microplastics as an emerging threat to terrestrial ecosystems. Glob. Chang. Biol. 24, 1405–1416. doi: 10.1111/gcb.14020, PMID: 29245177 PMC5834940

[ref23] de Souza Machado A. A. Lau C. W. Kloas W. Bergmann J. Bachelier J. B. Faltin E. . (2019). Microplastics can change soil properties and affect plant performance. Environ. Sci. Technol. 53, 6044–6052. doi: 10.1021/acs.est.9b01339, PMID: 31021077

[ref24] de Souza Machado A. A. Lau C. W. Till J. Kloas W. Lehmann A. Becker R. . (2018b). Impacts of microplastics on the soil biophysical environment. Environ. Sci. Technol. 52, 9656–9665. doi: 10.1021/acs.est.8b02212, PMID: 30053368 PMC6128618

[ref25] Delgado-Baquerizo M. Maestre F. T. Reich P. B. Jeffries T. C. Gaitan J. J. Encinar D. . (2016). Microbial diversity drives multifunctionality in terrestrial ecosystems. Nat. Commun. 7:10541. doi: 10.1038/ncomms10541, PMID: 26817514 PMC4738359

[ref26] Ding L. Huang D. Ouyang Z. Guo X. (2022). The effects of microplastics on soil ecosystem: a review. Curr Opin Environ Sci Health 26:100344. doi: 10.1016/j.coesh.2022.100344

[ref27] Ding J. Lv M. Zhu D. Leifheit E. F. Chen Q. L. Wang Y. Q. . (2022). Tire wear particles: An emerging threat to soil health. Crit Rev Env Sci Tec 53, 239–257.

[ref28] Dong Y. Gao M. Cai Q. Qiu W. Xiao L. Chen Z. . (2024b). The impact of microplastics on sulfur REDOX processes in different soil types: a mechanism study. J. Hazard. Mater. 465:133432. doi: 10.1016/j.jhazmat.2024.133432, PMID: 38219596

[ref29] Dong Y. Gao M. Qiu W. Xiao L. Cheng Z. Peng H. . (2024a). Investigating the impact of microplastics on sulfur mineralization in different soil types: a mechanism study. J. Hazard. Mater. 464:132942. doi: 10.1016/j.jhazmat.2023.132942, PMID: 37992502

[ref30] Drenovsky R. E. Vo D. Graham K. J. Scow K. M. (2004). Soil water content and organic carbon availability are major determinants of soil microbial community composition. Microb. Ecol. 48, 424–430. doi: 10.1007/s00248-003-1063-2, PMID: 15692862

[ref31] Fei Y. Huang S. Zhang H. Tong Y. Wen D. Xia X. . (2020). Response of soil enzyme activities and bacterial communities to the accumulation of microplastics in an acid cropped soil. Sci. Total Environ. 707:135634. doi: 10.1016/j.scitotenv.2019.135634 31761364

[ref32] Feng X. Wang Q. Sun Y. Zhang S. Wang F. (2022). Microplastics change soil properties, heavy metal availability and bacterial community in a Pb-Zn-contaminated soil. J. Hazard. Mater. 424:127364. doi: 10.1016/j.jhazmat.2021.127364, PMID: 34879561

[ref33] Fierer N. Jackson R. B. (2006). The diversity and biogeography of soil bacterial communities. P Natl Acad Sci USA 103, 626–631. doi: 10.1073/pnas.0507535103 PMC1334650 16407148

[ref34] Fu H. Z. Ho Y. S. Sui Y. M. Li Z. S. (2010). A bibliometric analysis of solid waste research during the period 1993-2008. Waste Manag. 30, 2410–2417. doi: 10.1016/j.wasman.2010.06.008, PMID: 20620038

[ref35] Fu F. Sun Y. Yang D. Zhao L. Li X. Weng L. . (2024). Combined pollution and soil microbial effect of pesticides and microplastics in greenhouse soil of suburban Tianjin. Northern China. Environ Pollut 340:122898. doi: 10.1016/j.envpol.2023.122898, PMID: 37944885

[ref36] Fuller S. Gautam A. (2016). A procedure for measuring microplastics using pressurized fluid extraction. Environ. Sci. Technol. 50, 5774–5780. doi: 10.1021/acs.est.6b00816, PMID: 27172172

[ref37] Gao H. Ding X. H. Wu S. (2020). Exploring the domain of open innovation: bibliometric and content analyses. J. Clean. Prod. 275:122580. doi: 10.1016/j.jclepro.2020.122580

[ref38] Gao Q. Lu X. Li J. Wang P. Li M. (2024). Impact of microplastics on nicosulfuron accumulation and bacteria community in soil-earthworms system. J. Hazard. Mater. 465:133414. doi: 10.1016/j.jhazmat.2023.133414 38181595

[ref39] Gao B. Yao H. Li Y. Zhu Y. (2021). Microplastic addition alters the microbial community structure and stimulates soil carbon dioxide emissions in vegetable-growing soil. Environ. Toxicol. Chem. 40, 352–365. doi: 10.1002/etc.4916, PMID: 33105038

[ref40] Geng Y. Zhu R. Maimaituerxun M. (2022). Bibliometric review of carbon neutrality with cite space: evolution, trends, and framework. Environ. Sci. Pollut. R. 29, 76668–76686. doi: 10.1007/s11356-022-23283-3, PMID: 36169840

[ref41] Grauwin S. Sperano I. (2018). Bibliomaps-a software to create web-based interactive maps of science: the case of UX map. Proc. Assoc. Inf. Sci. Technol. 55, 815–816. doi: 10.1002/pra2.2018.14505501129

[ref42] Guo J. J. Huang X. P. Xiang L. Wang Y. Z. Li Y. W. Li H. . (2020). Source, migration and toxicology of microplastics in soil. Environ. Int. 137:105263. doi: 10.1016/j.envint.2019.105263, PMID: 32087481

[ref43] Guo Z. Li P. Ma L. Yang X. Yang J. Wu Y. . (2024). Cascading effects from soil to maize functional traits explain maize response to microplastics disturbance in multi-nutrient soil environment. Geoderma 441:116759.

[ref44] Guo Z. Li P. Yang X. Wang Z. Lu B. Chen W. . (2022). Soil texture is an important factor determining how microplastics affect soil hydraulic characteristics. Environ. Int. 165:107293. doi: 10.1016/j.envint.2022.107293, PMID: 35609499

[ref45] Hanif M. N. Aijaz N. Azam K. Akhtar M. Laftah W. A. Babur M. . (2024). Impact of microplastics on soil (physical and chemical) properties, soil biological properties/soil biota, and response of plants to it: a review. Int. J. Environ. Sci. Technol., 1–42. doi: 10.1007/s13762-024-05656-y

[ref46] He D. Luo Y. Lu S. Liu M. Song Y. Lei L. (2018). Microplastics in soils: analytical methods, pollution characteristics and ecological risks. TrAC-Trend Anal Chem 109, 163–172. doi: 10.1016/j.trac.2018.10.006

[ref47] Hu X. Gu H. Wang Y. Liu J. Yu Z. Li Y. . (2022). Succession of soil bacterial communities and network patterns in response to conventional and biodegradable microplastics: a microcosmic study in Mollisol. J. Hazard. Mater. 436:129218. doi: 10.1016/j.jhazmat.2022.129218, PMID: 35739740

[ref48] Huang Y. Liu Q. Jia W. Yan C. Wang J. (2020). Agricultural plastic mulching as a source of microplastics in the terrestrial environment. Environ. Pollut. 260:114096. doi: 10.1016/j.envpol.2020.114096, PMID: 32041035

[ref49] Huang P. Zhang Y. Hussain N. Lan T. Chen G. Tang X. . (2024). A bibliometric analysis of global research hotspots and progress on microplastics in soil–plant systems. Environ. Pollut. 341:122890. doi: 10.1016/j.envpol.2023.122890, PMID: 37944892

[ref50] Huang Y. Zhao Y. Wang J. Zhang M. Jia W. Qin X. (2019). LDPE microplastic films alter microbial community composition and enzymatic activities in soil. Environ. Pollut. 254:112983. doi: 10.1016/j.envpol.2019.112983, PMID: 31394342

[ref51] Huffer T. Metzelder F. Sigmund G. Slawek S. Schmidt T. C. Hofmann T. (2019). Polyethylene microplastics influence the transport of organic contaminants in soil. Sci. Total Environ. 657, 242–247. doi: 10.1016/j.scitotenv.2018.12.047, PMID: 30543972

[ref52] Hummel D. Fath A. Hofmann T. Hüffer T. (2021). Additives and polymer composition influence the interaction of microplastics with xenobiotics. Environ. Chem. 18, 101–110. doi: 10.1071/EN21030

[ref53] Iqbal S. Xu J. Arif M. S. Shakoor A. Worthy F. R. Gui H. . (2024a). Could soil microplastic pollution exacerbate climate change? A meta-analysis of greenhouse gas emissions and global warming potential. Environ. Res. 252:118945. doi: 10.1016/j.envres.2024.118945, PMID: 38631466

[ref54] Iqbal S. Xu J. Arif M. S. Worthy F. R. Jones D. L. Khan S. . (2024b). Native soil properties, and prevailing climatic conditions have consequences for carbon and nitrogen contents in soil? A global data synthesis of pot and greenhouse studies. Environ. Sci. Technol. 58, 8464–8479. doi: 10.1021/acs.est.3c10247, PMID: 38701232

[ref55] Jia Y. Cheng Z. Peng Y. Yang G. (2024). Microplastics alter the equilibrium of plant-soil-microbial system: a meta-analysis. Ecotox Environ. Safety 272:116082. doi: 10.1016/j.ecoenv.2024.116082, PMID: 38335576

[ref56] Jiang X. Chen H. Liao Y. Ye Z. Li M. Klobucar G. (2019). Ecotoxicity and genotoxicity of polystyrene microplastics on higher plant *Vicia faba*. Environ. Pollut. 250, 831–838. doi: 10.1016/j.envpol.2019.04.055 31051394

[ref57] Khalid N. Aqeel M. Noman A. (2020). Microplastics could be a threat to plants in terrestrial systems directly or indirectly. Environ Pollut. 267:115653. doi: 10.1016/j.envpol.2020.115653, PMID: 33254725

[ref9001] Khalid N. Aqeel M. Noman A. Fatima Rizvi Z. (2023). Impact of plastic mulching as a major source of microplastics in agroecosystems. J. Hazard. Mater. 445:130455. doi: 10.1016/j.jhazmat.2022.130455, PMID: 36463747

[ref58] Kumar M. Xiong X. He M. Tsang D. C. W. Gupta J. Khan E. . (2020). Microplastics as pollutants in agricultural soils. Environ. Pollut. 265:114980. doi: 10.1016/j.envpol.2020.114980 32544663

[ref59] Kwak J. I. Kim L. An Y. J. (2024). Microplastics promote the accumulation of negative fungal groups and cause multigenerational effects in springtails. J. Hazard. Mater. 466:133574. doi: 10.1016/j.jhazmat.2024.133574 38280316

[ref60] Li J. Goerlandt F. Reniers G. (2021). An overview of scientometric mapping for the safety science community: methods, tools, and framework. Saf. Sci. 134:105093. doi: 10.1016/j.ssci.2020.105093

[ref61] Li K. Jia W. Xu L. Zhang M. Huang Y. (2023). The plastisphere of biodegradable and conventional microplastics from residues exhibit distinct microbial structure, network and function in plastic-mulching farmland. J. Hazard. Mater. 442:130011. doi: 10.1016/j.jhazmat.2022.130011, PMID: 36155295

[ref62] Li K. Ma D. Wu J. Chai C. Shi Y. (2016). Distribution of phthalate esters in agricultural soil with plastic film mulching in Shandong peninsula. East China. Chemosphere 164, 314–321. doi: 10.1016/j.chemosphere.2016.08.068, PMID: 27596820

[ref63] Li H. Q. Shen Y. J. Wang W. L. Wang H. T. Li H. Su J. Q. (2021). Soil pH has a stronger effect than arsenic content on shaping plastisphere bacterial communities in soil. Environ. Pollut. 287:117339. doi: 10.1016/j.envpol.2021.117339, PMID: 34000668

[ref64] Li Y. Shi X. Qin P. Zeng M. Fu M. Chen Y. . (2024). Effects of polyethylene microplastics and heavy metals on soil-plant microbial dynamics. Environ. Pollut. 341:123000. doi: 10.1016/j.envpol.2023.123000 38000728

[ref65] Li J. Zhang K. Zhang H. (2018). Adsorption of antibiotics on microplastics. Environ. Pollut. 237, 460–467. doi: 10.1016/j.envpol.2018.02.050 29510365

[ref66] Li H. Z. Zhu D. Lindhardt J. H. Lin S. M. Ke X. Cui L. (2021). Long-term fertilization history alters effects of microplastics on soil properties, microbial communities, and functions in diverse farmland ecosystem. Environ. Sci. Technol. 55, 4658–4668. doi: 10.1021/acs.est.0c04849, PMID: 33754703

[ref67] Liang Y. Lehmann A. Yang G. Leifheit E. F. Rillig M. C. (2021). Effects of microplastic fibers on soil aggregation and enzyme activities are organic matter dependent. Front Env Sci-Switz 9:650155. doi: 10.3389/fenvs.2021.650155

[ref68] Lin H. (2024). Bibliometric analysis of traffic-related air pollution: using cite space to explore the knowledge structure and trends. Environ Res Commun 6:022002. doi: 10.1088/2515-7620/ad2a92

[ref69] Liu X. Li Y. Yu Y. Yao H. (2023). Effect of nonbiodegradable microplastics on soil respiration and enzyme activity: a meta-analysis. Appl. Soil Ecol. 184:104770. doi: 10.1016/j.apsoil.2022.104770

[ref70] Liu M. Wang C. Zhu B. (2024). Independent and combined effects of microplastics pollution and drought on soil bacterial community. Sci. Total Environ. 913:169749. doi: 10.1016/j.scitotenv.2023.169749, PMID: 38160843

[ref71] Liu Z. Wen J. Liu Z. Wei H. Zhang J. (2024a). Polyethylene microplastics alter soil microbial community assembly and ecosystem multifunctionality. Environ. Int. 183:108360. doi: 10.1016/j.envint.2023.108360, PMID: 38128384

[ref72] Liu Z. Wu Z. Zhang Y. Wen J. Su Z. Wei H. . (2024b). Impacts of conventional and biodegradable microplastics in maize-soil ecosystems: above and below ground. J. Hazard. Mater. 477:135219. doi: 10.1016/j.jhazmat.2024.135129 39053066

[ref73] Lozano Y. M. Aguilar-Trigueros C. A. Onandia G. Maaß S. Zhao T. Rillig M. C. . (2021a). Effects of microplastics and drought on soil ecosystem functions and multifunctionality. J. Appl. Ecol. 58, 988–996. doi: 10.1111/1365-2664.13839

[ref74] Lozano Y. M. Dueñas J. F. Zordick C. Rillig M. C. (2024). Microplastic fibres affect soil fungal communities depending on drought conditions with consequences for ecosystem functions. Environ. Microbiol. 26:e16549. doi: 10.1111/1462-2920.16549, PMID: 38196372

[ref75] Lozano Y. M. Lehnert T. Linck L. T. Lehmann A. Rillig M. C. (2021b). Microplastic shape, polymer type, and concentration affect soil properties and plant biomass. Front. Plant Sci. 12:616645. doi: 10.3389/fpls.2021.616645, PMID: 33664758 PMC7920964

[ref76] Meng Q. Diao T. Yan L. Sun Y. (2023). Effects of single and combined contamination of microplastics and cadmium on soil organic carbon and microbial community structural: a comparison with different types of soil. Appl. Soil Ecol. 183:104763. doi: 10.1016/j.apsoil.2022.104763

[ref77] Moral-Muñoz J. A. Herrera-Viedma E. Santisteban-Espejo A. Cobo M. J. (2020). Software tools for conducting bibliometric analysis in science: An up-to-date review. El Profesional de la Información 29:e290103. doi: 10.3145/epi.2020.ene.03

[ref78] Nizzetto L. Futter M. Langaas S. (2016). Are agricultural soils dumps for microplastics of urban origin? Environ. Sci. Technol. 50, 10777–10779. doi: 10.1021/acs.est.6b04140, PMID: 27682621

[ref79] Paul J. Criado A. R. (2020). The art of writing literature review: what do we know and what do we need to know? Int. Bus. Rev. 29:101717. doi: 10.1016/j.ibusrev.2020.101717

[ref80] Perez-Reveron R. Gonzalez-Salamo J. Hernandez-Sanchez C. Gonzalez-Pleiter M. Hernandez-Borges J. Diaz-Pena F. J. (2022). Recycled wastewater as a potential source of microplastics in irrigated soils from an arid-insular territory (Fuerteventura, Spain). Sci. Total Environ. 817:152830. doi: 10.1016/j.scitotenv.2021.152830, PMID: 35016926

[ref81] Qi R. Jones D. L. Li Z. Liu Q. Yan C. (2020). Behavior of microplastics and plastic film residues in the soil environment: a critical review. Sci. Total Environ. 703:134722. doi: 10.1016/j.scitotenv.2019.134722, PMID: 31767311

[ref82] Qi R. Jones D. L. Tang Y. Gao F. Li J. Chi Y. . (2024). Regulatory path for soil microbial communities depends on the type and dose of microplastics. J. Hazard. Mater. 473:134702. doi: 10.1016/j.jhazmat.2024.134702, PMID: 38788589

[ref83] Qi Y. Ossowicki A. Yang X. Huerta Lwanga E. Dini-Andreote F. Geissen V. . (2020). Effects of plastic mulch film residues on wheat rhizosphere and soil properties. J. Hazard. Mater. 387:121711. doi: 10.1016/j.jhazmat.2019.121711, PMID: 31806445

[ref84] Qi Y. Yang X. Pelaez A. M. Huerta Lwanga E. Beriot N. Gertsen H. . (2018). Macro-and micro-plastics in soil-plant system: effects of plastic mulch film residues on wheat (*Triticum aestivum*) growth. Sci. Total Environ. 645, 1048–1056. doi: 10.1016/j.scitotenv.2018.07.229 30248830

[ref85] Qiu X. Qi Z. Ouyang Z. Liu P. Guo X. (2022). Interactions between microplastics and microorganisms in the environment: modes of action and influencing factors. Gondwana Res. 108, 102–119. doi: 10.1016/j.gr.2021.07.029

[ref86] Qiu Y. Zhou S. Zhang C. Zhou Y. Qin W. (2022). Soil microplastic characteristics and the effects on soil properties and biota: a systematic review and meta-analysis. Environ. Pollut. 313:120183. doi: 10.1016/j.envpol.2022.120183, PMID: 36126769

[ref87] Ranjbari M. Saidani M. Shams Esfandabadi Z. Peng W. Lam S. S. Aghbashlo M. . (2021). Two decades of research on waste management in the circular economy: insights from bibliometric, text mining, and content analyses. J. Clean. Prod. 314:128009. doi: 10.1016/j.jclepro.2021.128009

[ref88] Rauscher A. Meyer N. Jakobs A. Bartnick R. Lueders T. Lehndorff E. (2023). Biodegradable microplastic increases CO_2_ emission and alters microbial biomass and bacterial community composition in different soil types. Appl. Soil Ecol. 182:104714. doi: 10.1016/j.apsoil.2022.104714

[ref89] Rezaei M. Abbasi S. Pourmahmood H. Oleszczuk P. Ritsema C. Turner A. (2022). Microplastics in agricultural soils from a semi-arid region and their transport by wind erosion. Environ. Res. 212:113213. doi: 10.1016/j.envres.2022.113213, PMID: 35398314

[ref90] Rezaei M. Riksen M. Sirjani E. Sameni A. Geissen V. (2019). Wind erosion as a driver for transport of light density microplastics. Sci. Total Environ. 669, 273–281. doi: 10.1016/j.scitotenv.2019.02.382, PMID: 30878934

[ref91] Rillig M. C. de Souza Machado A. A. Lehmann A. Klumper U. (2019a). Evolutionary implications of microplastics for soil biota. Environ. Chem. 16, 3–7. doi: 10.1071/EN18118, PMID: 31231167 PMC6588528

[ref92] Rillig M. C. Lehmann A. de Souza Machado A. A. Yang G. (2019b). Microplastic effects on plants. New Phytol. 223, 1066–1070. doi: 10.1111/nph.15794 30883812

[ref93] Rochman C. M. Browne M. A. Underwood A. J. Van Franeker J. A. Thompson R. C. Amaral-Zettler L. A. (2016). The ecological impacts of marine debris: unraveling the demonstrated evidence from what is perceived. Ecology 97, 302–312. doi: 10.1890/14-2070.1, PMID: 27145606

[ref94] Romdhane S. Spor A. Aubert J. Bru D. Breuil M. C. Hallin S. . (2022). Unraveling negative biotic interactions determining soil microbial community assembly and functioning. ISME J. 16, 296–306. doi: 10.1038/s41396-021-01076-9, PMID: 34321619 PMC8692615

[ref95] Rüthi J. Bölsterli D. Pardi-Comensoli L. Brunner I. Frey B. (2020). The “Plastisphere” of biodegradable plastics is characterized by specific microbial taxa of alpine and Arctic soils. Front. Env. Sci. Switz 8:562263. doi: 10.3389/fenvs.2020.562263

[ref96] Salam M. Zheng H. Liu Y. Zaib A. Rehman S. A. U. Riaz N. . (2023). Effects of micro (nano) plastics on soil nutrient cycling: state of the knowledge. J. Environ. Manag. 344:118437. doi: 10.1016/j.jenvman.2023.118437, PMID: 37343476

[ref97] Schell T. Hurley R. Buenaventura N. T. Mauri P. V. Nizzetto L. Rico A. . (2022). Fate of microplastics in agricultural soils amended with sewage sludge: is surface water runoff a relevant environmental pathway? Environ. Pollut. 293:118520. doi: 10.1016/j.envpol.2021.118520 34800590

[ref98] Scheurer M. Bigalke M. (2018). Microplastics in Swiss floodplain soils. Environ. Sci. Technol. 52, 3591–3598. doi: 10.1021/acs.est.7b06003, PMID: 29446629

[ref99] Shen M. Huang W. Chen M. Song B. Zeng G. Zhang Y. (2020). (Micro) plastic crisis: un-ignorable contribution to global greenhouse gas emissions and climate change. J. Clean. Prod. 254:120138. doi: 10.1016/j.jclepro.2020.120138

[ref100] Shen M. Zhang Y. Zhu Y. Song B. Zeng G. Hu D. . (2019). Recent advances in toxicological research of nanoplastics in the environment: a review. Environ. Pollut. 252, 511–521. doi: 10.1016/j.envpol.2019.05.102, PMID: 31167159

[ref101] Shi J. Sun Y. Wang X. Wang J. (2022a). Microplastics reduce soil microbial network complexity and ecological deterministic selection. Environ. Microbiol. 24, 2157–2169. doi: 10.1111/1462-2920.15955, PMID: 35229440

[ref102] Shi J. Wang J. Lv J. Wang Z. Peng Y. Shang J. . (2022b). Microplastic additions alter soil organic matter stability and bacterial community under varying temperature in two contrasting soils. Sci. Total Environ. 838:156471. doi: 10.1016/j.scitotenv.2022.156471, PMID: 35660606

[ref103] Song X. Li C. Qiu Z. Wang C. Zeng Q. (2024). Ecotoxicological effects of polyethylene microplastics and lead (Pb) on the biomass, activity, and community diversity of soil microbes. Environ. Res. 252:119012. doi: 10.1016/j.envres.2024.119012, PMID: 38704010

[ref104] Steinmetz Z. Löffler P. Eichhöfer S. David J. Muñoz K. Schaumann G. E. (2022). Are agricultural plastic covers a source of plastic debris in soil? A first screening study. Soil 8, 31–47. doi: 10.5194/soil-8-31-2022

[ref105] Su P. Bu N. Liu X. Sun Q. Wang J. Zhang X. . (2024). Stimulated soil CO_2_ and CH_4_ emissions by microplastics: a hierarchical perspective. Soil Biol. Biochem. 194:109425. doi: 10.1016/j.soilbio.2024.109425

[ref106] Sun Y. Li X. Cao N. Duan C. Ding C. Huang Y. . (2022). Biodegradable microplastics enhance soil microbial network complexity and ecological stochasticity. J. Hazard. Mater. 439:129610. doi: 10.1016/j.jhazmat.2022.129610 35863232

[ref107] Sun J. Zhang X. Gong X. Sun Y. Zhang S. Wang F. (2024). Metagenomic analysis reveals gene taxonomic and functional diversity response to microplastics and cadmium in an agricultural soil. Environ. Res. 251:118673. doi: 10.1016/j.envres.2024.118673 38493845

[ref108] Sunil S. Bhagwat G. Vincent S. G. T. Palanisami T. (2024). Microplastics and climate change: the global impacts of a tiny driver. Sci. Total Environ. 946:174160. doi: 10.1016/j.scitotenv.2024.174160 38909818

[ref109] Temporiti M. E. E. Nicola L. Girometta C. E. Roversi A. Daccò C. Tosi S. (2022). The analysis of the mycobiota in plastic polluted soil reveals a reduction in metabolic ability. J Fung 8:1247. doi: 10.3390/jof8121247, PMID: 36547580 PMC9785340

[ref110] Thompson R. C. Olsen Y. Mitchell R. P. Davis A. Rowland S. J. John A. W. . (2004). Lost at sea: where is all the plastic? Science 304:838. doi: 10.1126/science.1094559 15131299

[ref111] Thor A. Marx W. Leydesdorff L. Bornmann L. (2016). Introducing cited references explorer (CRExplorer): a program for reference publication year spectroscopy with cited references standardization. J. Informetr. 10, 503–515. doi: 10.1016/j.joi.2016.02.005

[ref112] Tiwari N. Bansal M. Santhiya D. Sharma J. G. (2022). Insights into microbial diversity on plastisphere by multi-omics. Arch. Microb. 204:216. doi: 10.1007/s00203-022-02806-z 35316402

[ref113] van den Berg P. Huerta-Lwanga E. Corradini F. Geissen V. (2020). Sewage sludge application as a vehicle for microplastics in eastern Spanish agricultural soils. Environ. Pollut. 261:114198. doi: 10.1016/j.envpol.2020.114198, PMID: 32097788

[ref114] van Eck N. J. Waltman L. (2010). Software survey: VOSviewer, a computer program for bibliometric mapping. Scientometrics 84, 523–538. doi: 10.1007/s11192-009-0146-3 20585380 PMC2883932

[ref115] Wagg C. Bender S. F. Widmer F. van der Heijden M. G. (2014). Soil biodiversity and soil community composition determine ecosystem multifunctionality. Proc. Natl. Acad. Sci. USA 111, 5266–5270. doi: 10.1073/pnas.1320054111, PMID: 24639507 PMC3986181

[ref116] Wahyuningrum I. F. S. Humaira N. G. Budihardjo M. A. Arumdani I. S. Puspita A. S. Annisa A. N. . (2023). Environmental sustainability disclosure in Asian countries: bibliometric and content analysis. J. Clean. Prod. 411:137195. doi: 10.1016/j.jclepro.2023.137195

[ref117] Wang Q. Adams C. A. Wang F. Sun Y. Zhang S. (2021). Interactions between microplastics and soil fauna: a critical review. Crit. Rev. Env. Sci. Tec. 52, 3211–3243. doi: 10.1080/10643389.2021.1915035

[ref118] Wang W. Dong X. Qu J. Lin Y. Liu L. (2021). Bibliometric analysis of Microtia-related publications from 2006 to 2020. Ent-Ear Nose Throat 103, 36–40. doi: 10.1177/01455613211037641 34337975

[ref119] Wang P. Liu J. Han S. Wang Y. Duan Y. Liu T. . (2023). Polyethylene mulching film degrading bacteria within the plastisphere: co-culture of plastic degrading strains screened by bacterial community succession. J. Hazard. Mater. 442:130045. doi: 10.1016/j.jhazmat.2022.130045, PMID: 36162306

[ref120] Wang C. Wang L. Ok Y. S. Tsang D. C. W. Hou D. (2022). Soil plastisphere: exploration methods, influencing factors, and ecological insights. J. Hazard. Mater. 430:128503. doi: 10.1016/j.jhazmat.2022.128503, PMID: 35739682

[ref121] Wang L. Wu W. M. Bolan N. S. Tsang D. C. Li Y. Qin M. . (2021). Environmental fate, toxicity and risk management strategies of nanoplastics in the environment: current status and future perspectives. J. Hazard. Mater. 401:123415. doi: 10.1016/j.jhazmat.2020.123415, PMID: 32763705 PMC7345412

[ref122] Wang F. Zhang X. Zhang S. Zhang S. Sun Y. (2020). Interactions of microplastics and cadmium on plant growth and arbuscular mycorrhizal fungal communities in an agricultural soil. Chemosphere 254:126791. doi: 10.1016/j.chemosphere.2020.126791, PMID: 32320834

[ref123] Wei H. Wu L. Liu Z. Saleem M. Chen X. Xie J. . (2022). Meta-analysis reveals differential impacts of microplastics on soil biota. Ecotox Environ Safety 230:113150. doi: 10.1016/j.ecoenv.2021.113150, PMID: 34999340

[ref124] Weithmann N. Möller J. N. Löder M. G. J. Piehl S. Laforsch C. Freitag R. (2018). Organic fertilizer as a vehicle for the entry of microplastic into the environment. Sci. Adv. 4:eaap8060. doi: 10.1126/sciadv.aap8060, PMID: 29632891 PMC5884690

[ref125] Wu J. Y. Gao J. M. Pei Y. Z. Luo K. Y. Yang W. H. Wu J. C. . (2024). Microplastics in agricultural soils: a comprehensive perspective on occurrence, environmental behaviors and effects. Chem. Eng. J. 489:151328. doi: 10.1016/j.cej.2024.151328

[ref126] Wu Z. Kang L. Man Q. Xu X. Zhu F. Lyu H. (2024). Effects of hexabromocyclododecane and polyethylene microplastics on soil bacterial communities. Sci. Total Environ. 906:167691. doi: 10.1016/j.scitotenv.2023.167691, PMID: 37827321

[ref127] Wu C. Song X. Wang D. Ma Y. Shan Y. Ren X. . (2024). Combined effects of mulch film-derived microplastics and pesticides on soil microbial communities and element cycling. J. Hazard. Mater. 466:133656. doi: 10.1016/j.jhazmat.2024.133656, PMID: 38306832

[ref128] Xiao M. Ding J. Luo Y. Zhang H. Yu Y. Yao H. . (2022). Microplastics shape microbial communities affecting soil organic matter decomposition in paddy soil. J. Hazard. Mater. 431:128589. doi: 10.1016/j.jhazmat.2022.128589 35247738

[ref129] Xu M. Du W. Ai F. Xu F. Zhu J. Yin Y. . (2021). Polystyrene microplastics alleviate the effects of sulfamethazine on soil microbial communities at different CO_2_ concentrations. J. Hazard. Mater. 413:125286. doi: 10.1016/j.jhazmat.2021.125286 33592488

[ref130] Xu M. Xu Q. Wang G. Du W. Zhu J. Yin Y. . (2023). Elevated CO_2_ aggravated polystyrene microplastics effects on the rice-soil system under field conditions. Environ. Pollut. 316:120603. doi: 10.1016/j.envpol.2022.120603 36343858

[ref131] Ya H. Jiang B. Xing Y. Zhang T. Lv M. Wang X. (2021). Recent advances on ecological effects of microplastics on soil environment. Sci. Total Environ. 798:149338. doi: 10.1016/j.scitotenv.2021.149338, PMID: 34375233

[ref132] Ya H. Zhang T. Xing Y. Lv M. Wang X. Jiang B. (2023). Co-existence of polyethylene microplastics and tetracycline on soil microbial community and ARGs. Chemosphere 335:139082. doi: 10.1016/j.chemosphere.2023.139082, PMID: 37285974

[ref133] Yang M. Huang D. Y. Tian Y. B. Zhu Q. H. Zhang Q. Zhu H. H. . (2021). Influences of different source microplastics with different particle sizes and application rates on soil properties and growth of Chinese cabbage (*Brassica chinensis* L.). Ecotox Environ. Safety 222:112480. doi: 10.1016/j.ecoenv.2021.112480 34217116

[ref134] Yang B. Li P. Entemake W. Guo Z. Xue S. (2022). Concentration-dependent impacts of microplastics on soil nematode community in bulk soils of maize: evidence from a pot experiment. Front. Env. Sci. Switz 10:872898. doi: 10.3389/fenvs.2022.872898

[ref135] Yang J. Li L. Li R. Xu L. Shen Y. Li S. . (2021). Microplastics in an agricultural soil following repeated application of three types of sewage sludge: a field study. Environ. Pollut. 289:117943. doi: 10.1016/j.envpol.2021.117943, PMID: 34426179

[ref136] Yang Y. Li T. Liu P. Li H. Hu F. (2022). The formation of specific bacterial communities contributes to the enrichment of antibiotic resistance genes in the soil plastisphere. J. Hazard. Mater. 436:129247. doi: 10.1016/j.jhazmat.2022.129247, PMID: 35739766

[ref137] Ye N. Kueh T. B. Hou L. Liu Y. Yu H. (2020). A bibliometric analysis of corporate social responsibility in sustainable development. J. Clean. Prod. 272:122679. doi: 10.1016/j.jclepro.2020.122679

[ref138] Yu D. Chen Y. (2021). The analysis of the characteristics and evolution of the collaboration network in blockchain domain. Inform. Lithuan 32, 397–424. doi: 10.15388/20-INFOR437

[ref139] Yu H. Fan P. Hou J. Dang Q. Cui D. Xi B. . (2020). Inhibitory effect of microplastics on soil extracellular enzymatic activities by changing soil properties and direct adsorption: An investigation at the aggregate-fraction level. Environ. Pollut. 267:115544. doi: 10.1016/j.envpol.2020.115544, PMID: 32911337

[ref140] Zhang Y. Chen Y. (2020). Research trends and areas of focus on the Chinese loess plateau: a bibliometric analysis during 1991–2018. Catena 194:104798. doi: 10.1016/j.catena.2020.104798

[ref141] Zhang S. Han B. Sun Y. Wang F. (2020). Microplastics influence the adsorption and desorption characteristics of cd in an agricultural soil. J. Hazard. Mater. 388:121775. doi: 10.1016/j.jhazmat.2019.121775, PMID: 31813687

[ref142] Zhang H. Huang Y. An S. Zhu Z. (2022). A review of microplastics in soil: distribution within pedosphere compartments, environmental fate, and effects. Water Air Soil Poll. 233:380. doi: 10.1007/s11270-022-05837-w

[ref143] Zhang C. Lei Y. Qian J. Qiao Y. Liu J. Li S. . (2021). Sorption of organochlorine pesticides on polyethylene microplastics in soil suspension. Ecotoxicol. Environ. Safe 223:112591. doi: 10.1016/j.ecoenv.2021.112591, PMID: 34364123

[ref144] Zhang Z. Li Y. Qiu T. Duan C. Chen L. Zhao S. . (2022). Microplastics addition reduced the toxicity and uptake of cadmium to *Brassica chinensis* L. Sci. Total Environ. 852:158353. doi: 10.1016/j.scitotenv.2022.158353 36055513

[ref145] Zhang Y. Li X. Xiao M. Feng Z. Yu Y. Yao H. (2022). Effects of microplastics on soil carbon dioxide emissions and the microbial functional genes involved in organic carbon decomposition in agricultural soil. Sci. Total Environ. 806:150714. doi: 10.1016/j.scitotenv.2021.150714 34606872

[ref146] Zhang J. Li Z. Zhou X. Ding W. Wang X. Zhao M. . (2023). Long-term application of organic compost is the primary contributor to microplastic pollution of soils in a wheat-maize rotation. Sci. Total Environ. 866:161123. doi: 10.1016/j.scitotenv.2022.161123, PMID: 36586695

[ref147] Zhang S. Pei L. Zhao Y. Shan J. Zheng X. Xu G. . (2023). Effects of microplastics and nitrogen deposition on soil multifunctionality, particularly C and N cycling. J. Hazard. Mater. 451:131152. doi: 10.1016/j.jhazmat.2023.131152 36934700

[ref148] Zhang T. Tang Y. Li H. Hu W. Cheng J. Lee X. (2023). A bibliometric review of biochar for soil carbon sequestration and mitigation from 2001 to 2020. Ecotox Environ. Safe 264:115438. doi: 10.1016/j.ecoenv.2023.115438, PMID: 37683427

[ref149] Zhang Z. Wang W. Liu J. Wu H. (2024). Discrepant responses of bacterial community and enzyme activities to conventional and biodegradable microplastics in paddy soil. Sci. Total Environ. 909:168513. doi: 10.1016/j.scitotenv.2023.168513 37977392

[ref150] Zhang L. Xie Y. Liu J. Zhong S. Qian Y. Gao P. (2020). An overlooked entry pathway of microplastics into agricultural soils from application of sludge-based fertilizers. Environ. Sci. Technol. 54, 4248–4255. doi: 10.1021/acs.est.9b07905, PMID: 32131589

[ref151] Zhang B. Yang X. Chen L. Chao J. Teng J. Wang Q. (2020). Microplastics in soils: a review of possible sources, analytical methods and ecological impacts. J. Chem. Technol. Biot. 95, 2052–2068. doi: 10.1002/jctb.6334

[ref152] Zhang M. Zhao Y. Qin X. Jia W. Chai L. Huang M. . (2019). Microplastics from mulching film is a distinct habitat for bacteria in farmland soil. Sci. Total Environ. 688, 470–478. doi: 10.1016/j.scitotenv.2019.06.108, PMID: 31254812

[ref153] Zhao Z. Y. Wang P. Y. Wang Y. B. Zhou R. Koskei K. Munyasya A. N. . (2021). Fate of plastic film residues in agro-ecosystem and its effects on aggregateassociated soil carbon and nitrogen stocks. J. Hazard. Mater. 416:125954. doi: 10.1016/j.jhazmat.2021.125954, PMID: 34492872

[ref154] Zhou Z. Hua J. Xue J. Yu C. (2024). Differential impacts of polyethylene microplastic and additives on soil nitrogen cycling: a deeper dive into microbial interactions and transformation mechanisms. Sci. Total Environ. 942:173771. doi: 10.1016/j.scitotenv.2024.173771, PMID: 38851351

[ref155] Zhou B. Wang J. Zhang H. Shi H. Fei Y. Huang S. . (2020). Microplastics in agricultural soils on the coastal plain of Hangzhou Bay, East China: multiple sources other than plastic mulching film. J. Hazard. Mater. 388:121814. doi: 10.1016/j.jhazmat.2019.121814 31843412

[ref156] Zhou Y. Wang J. Zou M. Jia Z. Zhou S. Li Y. (2020). Microplastics in soils: a review of methods, occurrence, fate, transport, ecological and environmental risks. Sci. Total Environ. 748:141368. doi: 10.1016/j.scitotenv.2020.141368, PMID: 32798871

[ref157] Zhou J. Zeng A. Fan Y. Di Z. (2018). Identifying important scholars via directed scientific collaboration networks. Scientometrics 114, 1327–1343. doi: 10.1007/s11192-017-2619-0

[ref158] Zhu D. Chen Q. L. An X. L. Yang X. R. Christie P. Ke X. . (2018). Exposure of soil collembolans to microplastics perturbs their gut microbiota and alters their isotopic composition. Soil Biol. Biochem. 116, 302–310. doi: 10.1016/j.soilbio.2017.10.027

[ref159] Zhu D. Ma J. Li G. Rillig M. C. Zhu Y. G. (2022). Soil plastispheres as hotpots of antibiotic resistance genes and potential pathogens. ISME J. 16, 521–532. doi: 10.1038/s41396-021-01103-9, PMID: 34455424 PMC8776808

[ref160] Zhu F. Yan Y. Doyle E. Zhu C. Jin X. Chen Z. . (2022). Microplastics altered soil microbiome and nitrogen cycling: the role of phthalate plasticizer. J. Hazard. Mater. 427:127944. doi: 10.1016/j.jhazmat.2021.127944, PMID: 34865900

