# Supplementary material for: Global perspective of ecological risk of plastic pollution on soil microbial communities
Source: Front Microbiol. 2024 Oct 9;15:1468592. doi: 10.3389/fmicb.2024.1468592 (PMC11496196; doi:10.3389/fmicb.2024.1468592)
Supplement: Supplementary file 1 [file Table_1.docx]

**Table S1**. Main information about retrieval documents used to bibliometic analysis

| Description | Results |
| --- | --- |
| MAIN INFORMATION ABOUT DATA |  |
| Timespan | 2011:2023 |
| Sources (Journals, Books, etc) | 122 |
| Documents | 452 |
| Annual Growth Rate % | 19.38 |
| Document Average Age | 2.11 |
| Average citations per doc | 37.31 |
| References | 16618 |
| DOCUMENT CONTENTS |  |
| Keywords Plus (ID) | 823 |
| Author's Keywords (DE) | 1110 |
| AUTHORS |  |
| Authors | 2070 |
| Authors of single-authored docs | 2 |
| AUTHORS COLLABORATION |  |
| Single-authored docs | 2 |
| Co-Authors per Doc | 6.76 |
| International co-authorships % | 31.86 |
| DOCUMENT TYPES |  |
| article | 329 |
| article; early access | 7 |
| correction | 1 |
| editorial material | 1 |
| review | 108 |
| review; early access | 6 |
