# Supplementary material for: Global perspective of ecological risk of plastic pollution on soil microbial communities
Source: Front Microbiol. 2024 Oct 9;15:1468592. doi: 10.3389/fmicb.2024.1468592 (PMC11496196; doi:10.3389/fmicb.2024.1468592)
Supplement: Supplementary file 2 [file Table_2.docx]

**Table S2.** Characteristics of terms in co-occurrence analysis of keywords

| **Keywords** | **Frequency** | **Cluster** | **Degree** | **Centrality** | | **Sigma** | **Year** |
| --- | --- | --- | --- | --- | --- | --- | --- |
| soil microbial community | 58 | 0 | 16 | 0.23 | | 1 | 2019 |
| biodegradable microplastics | 16 | 0 | 7 | 0.23 | | 1 | 2020 |
| soil properties | 13 | 0 | 4 | 0.08 | | 1 | 2020 |
| heavy metals | 11 | 0 | 5 | 0.03 | | 1 | 2020 |
| co-occurrence network | 6 | 0 | 7 | 0.08 | | 1 | 2019 |
| arbuscular mycorrhizae | 5 | 0 | 6 | 0.12 | | 1 | 2020 |
| functional prediction | 3 | 0 | 3 | 0.04 | | 1 | 2019 |
| high-throughput sequencing | 3 | 0 | 3 | 0.03 | | 1 | 2019 |
| assembly process | 3 | 0 | 1 | 0 | | 1 | 2022 |
| combined effect | 3 | 0 | 1 | 0 | | 1 | 2019 |
| community assembly | 3 | 0 | 1 | 0 | | 1 | 2023 |
| mulching film | 1 | 0 | 3 | 0 | | 1 | 2019 |
| surface morphology | 1 | 0 | 3 | 0 | | 1 | 2019 |
| environmental concerns | 1 | 0 | 2 | 0 | | 1 | 2019 |
| biodiversity change | 1 | 0 | 2 | 0 | | 1 | 2019 |
| carbon content | 1 | 0 | 2 | 0 | | 1 | 2020 |
| earth system processes | 1 | 0 | 1 | 0 | | 1 | 2021 |
| alternate wetting and drying | 1 | 0 | 1 | 0 | | 1 | 2023 |
| anthropogenic activities | 1 | 0 | 1 | 0 | | 1 | 2022 |
| acid phosphatase | 1 | 0 | 1 | 0 | | 1 | 2020 |
| cadmium accumulation | 1 | 0 | 1 | 0 | | 1 | 2023 |
| CO_2_ emissions | 1 | 0 | 1 | 0 | | 1 | 2021 |
| biomarker taxa | 1 | 0 | 1 | 0 | | 1 | 2022 |
| ambient temperature | 1 | 0 | 1 | 0 | | 1 | 2023 |
| functional diversity | 1 | 0 | 1 | 0 | | 1 | 2021 |
| engineered nanoparticles | 1 | 0 | 1 | 0 | | 1 | 2021 |
| soil bacterial community | 23 | 1 | 5 | 0.04 | | 1 | 2020 |
| ecological risks | 14 | 1 | 6 | 0.15 | | 1 | 2018 |
| analytical methods | 11 | 1 | 10 | 0.28 | | 1 | 2018 |
| environmental pollution | 9 | 1 | 4 | 0.03 | | 1 | 2020 |
| terrestrial ecosystem | 6 | 1 | 7 | 0.15 | | 1 | 2018 |
| toxicity | 2 | 1 | 2 | 0.01 | | 1 | 2022 |
| crop production | 2 | 1 | 2 | 0.03 | | 1 | 2022 |
| aging effects | 2 | 1 | 1 | 0 | | 1 | 2023 |
| biological effect | 2 | 1 | 1 | 0 | | 1 | 2021 |
| greenhouse gases (GHGs) | 1 | 1 | 3 | 0.03 | | 1 | 2020 |
| pollution characteristics | 1 | 1 | 3 | 0 | | 1 | 2018 |
| fungi | 1 | 1 | 3 | 0.03 | | 1 | 2020 |
| ^15^ N fertilizer recovery | 1 | 1 | 2 | 0 | | 1 | 2023 |
| AM symbiosis | 1 | 1 | 2 | 0 | | 1 | 2023 |
| ecological surprise | 1 | 1 | 2 | 0 | | 1 | 2021 |
| ecosystem services | 1 | 1 | 2 | 0 | | 1 | 2021 |
| agricultural systems | 1 | 1 | 2 | 0 | | 1 | 2020 |
| critical review | 1 | 1 | 1 | 0 | | 1 | 2021 |
| biological sewage treatment | 1 | 1 | 1 | 0 | | 1 | 2022 |
| available phosphorus | 1 | 1 | 1 | 0 | | 1 | 2021 |
| aging process | 1 | 1 | 1 | 0 | | 1 | 2022 |
| biodegradable plastics | 22 | 2 | 9 | 0.18 | | 1 | 2018 |
| plastic mulch film | 18 | 2 | 10 | 0.3 | | 1 | 2016 |
| soil health | 12 | 2 | 13 | 0.36 | | 1 | 2018 |
| soil physicochemical properties | 10 | 2 | 14 | 0.3 | | 1 | 2018 |
| mitigation strategies | 2 | 2 | 1 | 0 | | 1 | 2023 |
| soil microbiology | 1 | 2 | 6 | 0 | | 1 | 2018 |
| specialty crops | 1 | 2 | 6 | 0 | | 1 | 2018 |
| soil microclimate | 1 | 2 | 6 | 0 | | 1 | 2018 |
| ecotoxicity monitoring | 1 | 2 | 3 | 0 | | 1 | 2021 |
| biodegradable plastic mulch | 1 | 2 | 3 | 0 | | 1 | 2021 |
| crop safety | 1 | 2 | 3 | 0 | | 1 | 2021 |
| aggregate-size fractions | 1 | 2 | 2 | 0 | | 1 | 2020 |
| carbon and nitrogen cycles | 1 | 2 | 2 | 0 | | 1 | 2023 |
| extracellular enzyme activities | 1 | 2 | 2 | 0 | | 1 | 2020 |
| carbon and nitrogen coupling | 1 | 2 | 2 | 0 | | 1 | 2023 |
| 16s rRNA | 1 | 2 | 1 | 0 | | 1 | 2023 |
| agricultural film | 1 | 2 | 1 | 0 | | 1 | 2023 |
| agricultural fields | 1 | 2 | 1 | 0 | | 1 | 2023 |
| antibiotic resistance gene reduction | 1 | 2 | 1 | 0 | | 1 | 2023 |
| biodegradable mulch | 1 | 2 | 1 | 0 | | 1 | 2023 |
| plastic pollution | 18 | 3 | 6 | 0.15 | | 1 | 2020 |
| polyethylene microplastics | 11 | 3 | 7 | 0.08 | | 1 | 2021 |
| microbial diversity | 8 | 3 | 4 | 0.06 | | 1 | 2021 |
| community structure | 6 | 3 | 3 | 0.03 | | 1 | 2019 |
| black soil | 2 | 3 | 2 | 0.04 | | 1 | 2020 |
| crop growth | 2 | 3 | 2 | 0.01 | | 1 | 2022 |
| nitrous oxide | 2 | 3 | 1 | 0 | | 1 | 2022 |
| FESEM imaging | 1 | 3 | 2 | 0.05 | | 1 | 2021 |
| cadmium (Cd) | 1 | 3 | 2 | 0 | | 1 | 2021 |
| combined toxicity | 1 | 3 | 2 | 0 | | 1 | 2021 |
| acid rain | 1 | 3 | 1 | 0 | | 1 | 2022 |
| biodegradable mulch residue | 1 | 3 | 1 | 0 | | 1 | 2022 |
| metabolism | 1 | 3 | 1 | 0 | | 1 | 2019 |
| biological impacts | 1 | 3 | 1 | 0 | | 1 | 2023 |
| acid deposition | 1 | 3 | 1 | 0 | | 1 | 2022 |
| belowground C input | 1 | 3 | 1 | 0 | | 1 | 2020 |
| bio-based and biodegradable plastic | 1 | 3 | 1 | 0 | | 1 | 2022 |
| available nitrogen | 1 | 3 | 1 | 0 | | 1 | 2023 |
| microplastic pollution | 19 | 4 | 6 | 0.2 | | 1 | 2020 |
| soil quality | 7 | 4 | 5 | 0.06 | | 1 | 2018 |
| ecosystem functioning | 6 | 4 | 5 | 0.12 | | 1 | 2020 |
| soil food web | 5 | 4 | 4 | 0.03 | | 1 | 2018 |
| belowground biodiversity | 1 | 4 | 3 | 0.02 | | 1 | 2020 |
| plastic degradation | 1 | 4 | 2 | 0 | | 1 | 2018 |
| food safety | 1 | 4 | 2 | 0.03 | | 1 | 2020 |
| plant response | 1 | 4 | 2 | 0 | | 1 | 2018 |
| pesticide decay | 1 | 4 | 2 | 0 | | 1 | 2018 |
| soil microbial activities | 1 | 4 | 2 | 0 | | 1 | 2018 |
| agroecosystem resilience | 1 | 4 | 2 | 0.07 | | 1 | 2022 |
| biogeographical pattern | 1 | 4 | 1 | 0 | | 1 | 2022 |
| carbon metabolism | 1 | 4 | 1 | 0 | | 1 | 2022 |
| C turnover | 1 | 4 | 1 | 0 | | 1 | 2021 |
| bacterial and fungal diversity | 1 | 4 | 1 | 0 | | 1 | 2022 |
| agrochemical degradation | 1 | 4 | 1 | 0 | | 1 | 2022 |
| soil aggregation | 7 | 5 | 6 | 0.19 | | 1 | 2019 |
| bacterial diversity | 6 | 5 | 5 | 0.06 | | 1 | 2022 |
| nutrient cycling | 4 | 5 | 7 | 0.12 | | 1 | 2021 |
| functional genes | 3 | 5 | 4 | 0.03 | | 1 | 2023 |
| soil respiration | 3 | 5 | 3 | 0 | | 1 | 2021 |
| plastic residues | 3 | 5 | 1 | 0 | | 1 | 2022 |
| soil pH | 2 | 5 | 4 | 0.09 | | 1 | 2021 |
| carbon sequestration | 2 | 5 | 3 | 0.01 | | 1 | 2023 |
| plant growth | 2 | 5 | 2 | 0.03 | | 1 | 2021 |
| rhizosphere soil | 2 | 5 | 1 | 0 | | 1 | 2023 |
| agricultural mulching film | 1 | 5 | 1 | 0 | | 1 | 2022 |
| ^13^ C stable isotope | 1 | 5 | 1 | 0 | | 1 | 2023 |
| carbon and nitrogen cycling | 1 | 5 | 1 | 0 | | 1 | 2023 |
| bacterial composition | 1 | 5 | 1 | 0 | | 1 | 2023 |
| soil enzyme activity | 46 | 6 | 9 | 0.18 | | 1 | 2016 |
| paddy soil | 6 | 6 | 5 | 0.07 | | 1 | 2019 |
| microbial biomass | 3 | 6 | 3 | 0.01 | | 1 | 2018 |
| food security | 2 | 6 | 2 | 0.01 | | 1 | 2022 |
| biodegradable seedling trays | 1 | 6 | 2 | 0 | | 1 | 2019 |
| environmental risk assessment | 1 | 6 | 2 | 0 | | 1 | 2018 |
| bacterial community turnover | 1 | 6 | 2 | 0 | | 1 | 2022 |
| carbon cycling | 1 | 6 | 2 | 0 | | 1 | 2022 |
| amino acid | 1 | 6 | 2 | 0.03 | | 1 | 2022 |
| anaerobic metabolic | 1 | 6 | 1 | 0 | | 1 | 2023 |
| antibiotics resistance | 1 | 6 | 1 | 0 | | 1 | 2022 |
| ammonia volatilization | 1 | 6 | 1 | 0 | | 1 | 2022 |
| C cinerea | 1 | 6 | 1 | 0 | | 1 | 2023 |
| different depths | 1 | 6 | 1 | 0 | | 1 | 2021 |
| microbes | 21 | 7 | 13 | 0.25 | | 1 | 2015 |
| low-density polyethylene | 3 | 7 | 2 | 0 | | 1 | 2022 |
| soil nutrients | 3 | 7 | 1 | 0 | | 1 | 2023 |
| gut microbiota | 2 | 7 | 2 | 0.01 | | 1 | 2021 |
| ecotoxicity | 2 | 7 | 1 | 0 | | 1 | 2020 |
| aliphatic-aromatic co-polyesters | 1 | 7 | 3 | 0.01 | | 1 | 2022 |
| biodegradation in soil | 1 | 7 | 3 | 0.01 | | 1 | 2022 |
| community dynamics | 1 | 7 | 3 | 0 | | 1 | 2015 |
| *Zea mays* | 1 | 7 | 3 | 0 | | 1 | 2015 |
| Illumina Miseq | 1 | 7 | 3 | 0 | | 1 | 2015 |
| wet-dry cycle | 1 | 7 | 2 | 0 | | 1 | 2019 |
| agriculture contamination | 1 | 7 | 1 | 0 | | 1 | 2023 |
| antioxidant enzymes | 1 | 7 | 1 | 0 | | 1 | 2021 |
| soil | 10 | 8 | 2 | 0.02 | | 1 | 2022 |
| soil biota | 9 | 8 | 4 | 0.07 | | 1 | 2022 |
| nitrogen cycling | 7 | 8 | 4 | 0.07 | | 1 | 2023 |
| climate change | 5 | 8 | 2 | 0.01 | | 1 | 2022 |
| heat waves | 2 | 8 | 2 | 0.03 | | 1 | 2023 |
| biogeochemical cycles | 2 | 8 | 1 | 0 | | 1 | 2020 |
| nitrogen uptake | 2 | 8 | 1 | 0 | | 1 | 2023 |
| biogeochemical cycling | 1 | 8 | 3 | 0.09 | | 1 | 2023 |
| biodiversity loss | 1 | 8 | 2 | 0.03 | | 1 | 2023 |
| C cycle | 1 | 8 | 1 | 0 | | 1 | 2023 |
| agricultural soils | 1 | 8 | 1 | 0 | | 1 | 2023 |
| artificial intelligence | 1 | 8 | 1 | 0 | | 1 | 2022 |
| soil organic carbon | 5 | 9 | 4 | 0.1 | | 1 | 2021 |
| soil carbon storage | 3 | 9 | 5 | 0.15 | | 1 | 2023 |
| dissolved organic carbon | 3 | 9 | 2 | 0.01 | | 1 | 2023 |
| carbon dioxide | 2 | 9 | 4 | 0.04 | | 1 | 2023 |
| degradable microplastics | 2 | 9 | 4 | 0.04 | | 1 | 2023 |
| biodegradable film | 1 | 9 | 2 | 0.03 | | 1 | 2023 |
| biodegradable plastic particle | 1 | 9 | 1 | 0 | | 1 | 2023 |
| biodegradable mulch films | 1 | 9 | 1 | 0 | | 1 | 2023 |
| biodegradable and non-biodegradable MPs | 1 | 9 | 1 | 0 | | 1 | 2023 |
| bacterial diversity and community | 1 | 9 | 1 | 0 | | 1 | 2023 |
| greenhouse gas emissions | 11 | 10 | 4 | 0.14 | | 1 | 2022 |
| agricultural ecosystems | 9 | 10 | 4 | 0.21 | | 1 | 2021 |
| emerging contaminants | 6 | 10 | 5 | 0.22 | | 1 | 2022 |
| antibiotic resistance genes | 5 | 10 | 2 | 0.01 | | 1 | 2019 |
| carbon and nitrogen turnover | 1 | 10 | 2 | 0.03 | | 1 | 2022 |
| agricultural ecosystem | 1 | 10 | 1 | 0 | 1 | | 2022 |
| carbon cycling | 1 | 10 | 1 | 0 | 1 | | 2023 |
| bisphenol s | 1 | 10 | 1 | 0 | 1 | | 2022 |
| aged microplastics | 1 | 10 | 1 | 0 | 1 | | 2023 |
| soil contamination | 6 | 11 | 2 | 0.01 | 1 | | 2020 |
| food chain | 5 | 11 | 2 | 0.01 | 1 | | 2022 |
| human health | 3 | 11 | 1 | 0 | 1 | | 2023 |
| bibliometric analysis | 2 | 11 | 1 | 0 | 1 | | 2023 |
| antibiotic resistant bacteria | 1 | 11 | 3 | 0.06 | 1 | | 2022 |
| water-stable aggregates | 2 | 12 | 2 | 0 | 1 | | 2021 |
| soil water status | 2 | 12 | 2 | 0 | 1 | | 2021 |
| *Daucus carota* | 2 | 12 | 2 | 0 | 1 | | 2021 |
| elevated CO_2_ | 1 | 13 | 1 | 0 | 1 | | 2021 |
| antibiotic-resistance genes | 1 | 13 | 1 | 0 | 1 | | 2021 |
| arctic soil | 1 | 14 | 1 | 0 | 1 | | 2020 |
| alpine soil | 1 | 14 | 1 | 0 | 1 | | 2020 |
| polymer degradation plate assays | 1 | 15 | 1 | 0 | 1 | | 2018 |
| poly(lactic acid)/poly(3-hydroxybutyrate) blend | 1 | 15 | 1 | 0 | 1 | | 2018 |
| bioplastic production | 1 | 16 | 1 | 0 | 1 | | 2020 |
| chemical recycling | 1 | 16 | 1 | 0 | 1 | | 2020 |
| waste management | 1 | 17 | 1 | 0 | 1 | | 2019 |
| ecosystem health | 1 | 17 | 1 | 0 | 1 | | 2019 |
| antibiotic resistome | 1 | 18 | 1 | 0 | 1 | | 2022 |
| bacterial community shift | 1 | 18 | 1 | 0 | 1 | | 2022 |
| biodegradable polymer | 1 | 19 | 0 | 0 | 1 | | 2020 |
| alkaline phosphatase | 1 | 20 | 0 | 0 | 1 | | 2023 |
| bacillus amyloliquefaciens | 1 | 21 | 0 | 0 | 1 | | 2022 |
| environmental risk | 1 | 22 | 0 | 0 | 1 | | 2020 |
| *Avena sativa* | 1 | 23 | 0 | 0 | 1 | | 2021 |
| *Alternaria alternata* | 1 | 24 | 0 | 0 | 1 | | 2015 |
| animal health | 1 | 25 | 0 | 0 | 1 | | 2021 |
| bacterial community assembly | 1 | 26 | 0 | 0 | 1 | | 2023 |
| ecoflex (r) | 1 | 27 | 0 | 0 | 1 | | 2016 |
| analytical methodology | 1 | 28 | 0 | 0 | 1 | | 2021 |
| agricultural plastic | 1 | 29 | 0 | 0 | 1 | | 2022 |
| black carbon | 1 | 30 | 0 | 0 | 1 | | 2023 |
| 16s RNA | 1 | 31 | 0 | 0 | 1 | | 2021 |
| 16s rRNA gene sequencing | 1 | 32 | 0 | 0 | 1 | | 2022 |
| biodegradable plastic mulches | 1 | 33 | 0 | 0 | 1 | | 2021 |
| ecotoxicological impacts | 1 | 34 | 0 | 0 | 1 | | 2020 |
| selection pressures | 1 | 35 | 0 | 0 | 1 | | 2019 |
| antimicrobial resistance | 1 | 36 | 0 | 0 | 1 | | 2023 |
| carbon cycle | 1 | 37 | 0 | 0 | 1 | | 2022 |
| health risks | 1 | 38 | 0 | 0 | 1 | | 2020 |
| rhizosphere microbial community | 4 | 39 | 0 | 0 | 1 | | 2022 |
| bacterial dynamics | 1 | 40 | 0 | 0 | 1 | | 2022 |
| Bin Chen | 1 | 41 | 0 | 0 | 1 | | 2022 |
| cell wall | 1 | 42 | 0 | 0 | 1 | | 2020 |
| microbial consortium | 1 | 43 | 0 | 0 | 1 | | 2015 |
| microbial electrochemical system | 2 | 44 | 0 | 0 | 1 | | 2023 |
