# Supplementary material for: Global perspective of ecological risk of plastic pollution on soil microbial communities
Source: Front Microbiol. 2024 Oct 9;15:1468592. doi: 10.3389/fmicb.2024.1468592 (PMC11496196; doi:10.3389/fmicb.2024.1468592)

**Figure S1.** Flow diagram of the bibliometric analysis of interactions between plastic pollution and soil microorganisms**.**


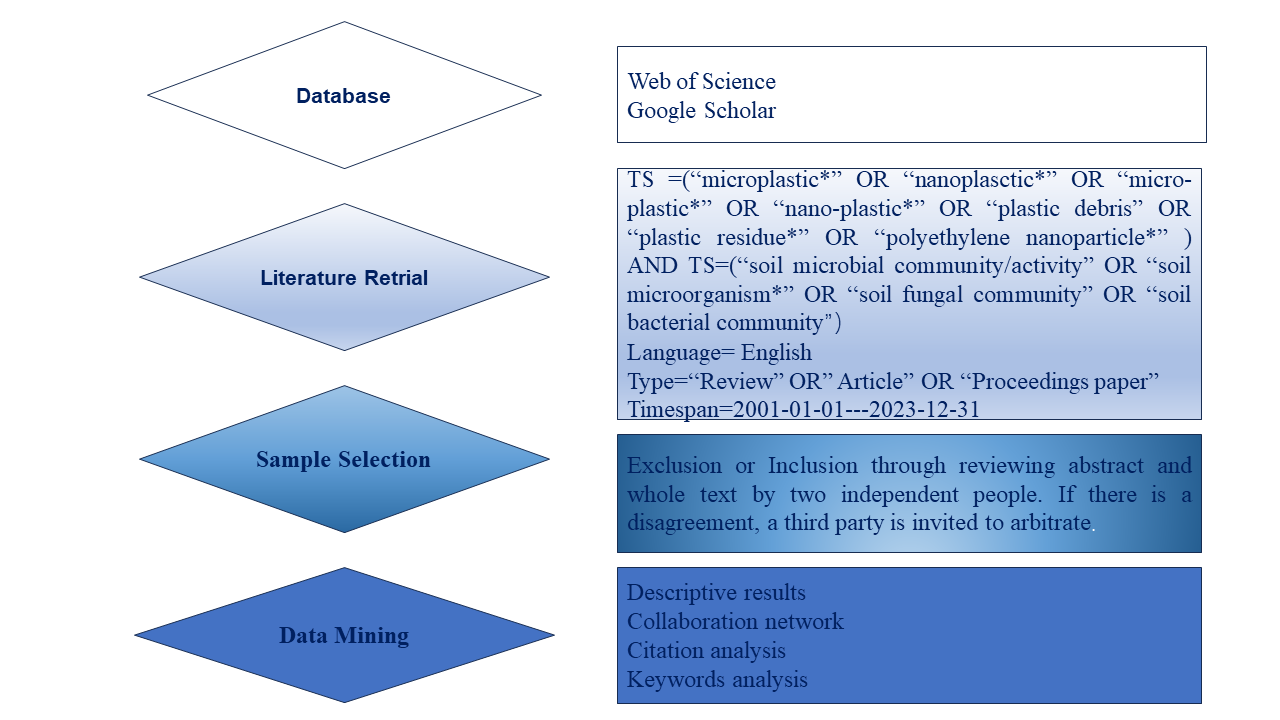


**Fig. S2** Thematic evolution of studies addressing the ecological impacts of plastic pollution on soil microbes. Thematic evolution of studies addressing the ecological impacts of plastic pollution on soil microbes during 2011-2023 (A). Each node represents a popular keyword, and the size of each node is proportional to its cited frequency. The line between each node represents the temporal evolution of the keyword; these lines reflect the relationship between transfer and inheritance among keywords. Thematic map of 2020 (B), 2021(C), 2022 (D) and 2023 (E) based on density and centrality, divided into four topological regions. The upper right quadrant shows “motor” or “driving” topics, indicated by high density and centrality; these topics should be developed further given their importance for future research. The quadrant in the top left shows specific and under-represented topics that nonetheless are areas of rapid development, as indicated by high density but low centrality. The lower left quadrant contains topics that have been used but have experienced a downward trend or that newly occurred, indicated by low centrality and density. The lower right quadrant contains basic topics, indicated by high centrality but low density; these topics are important for research as general topics.


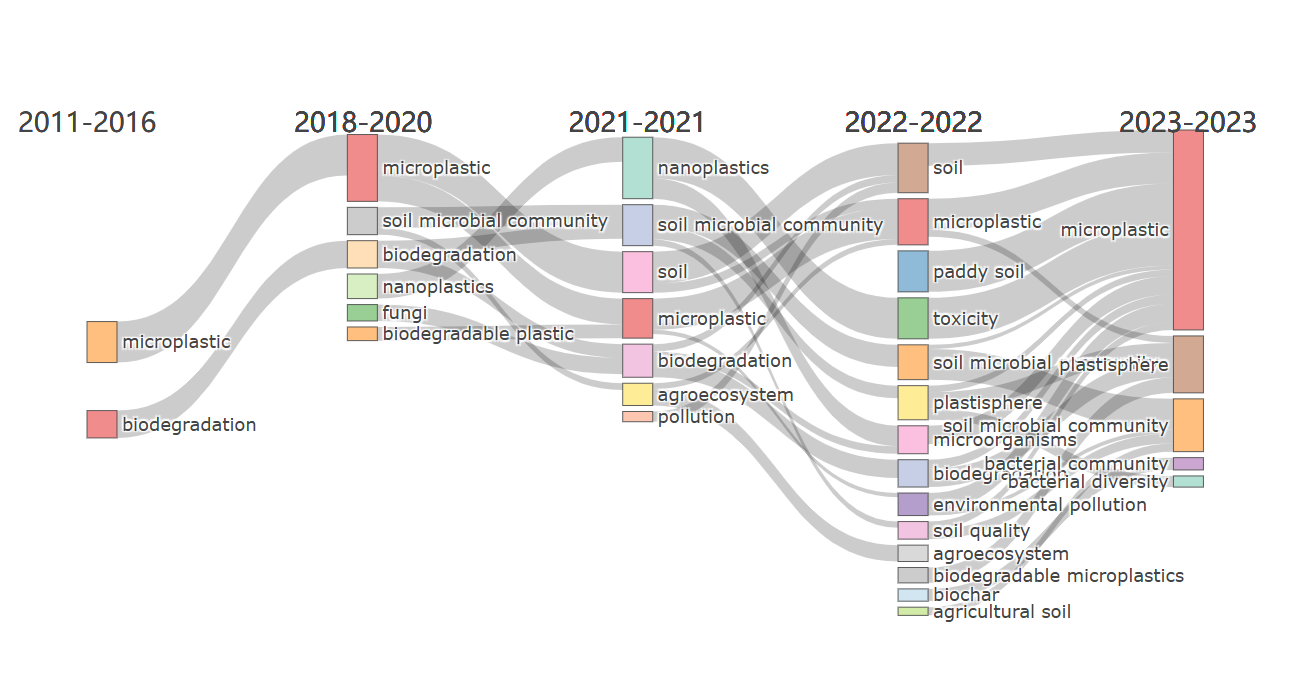


**(B)**


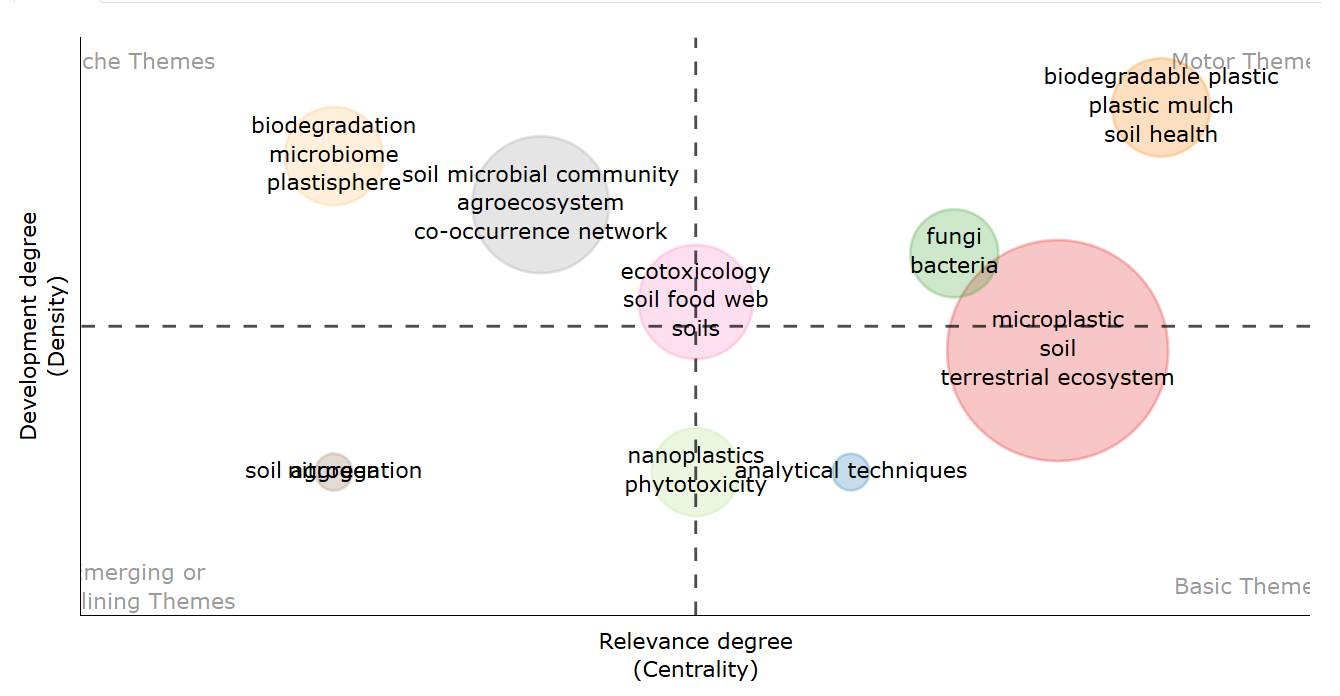


**(C)**


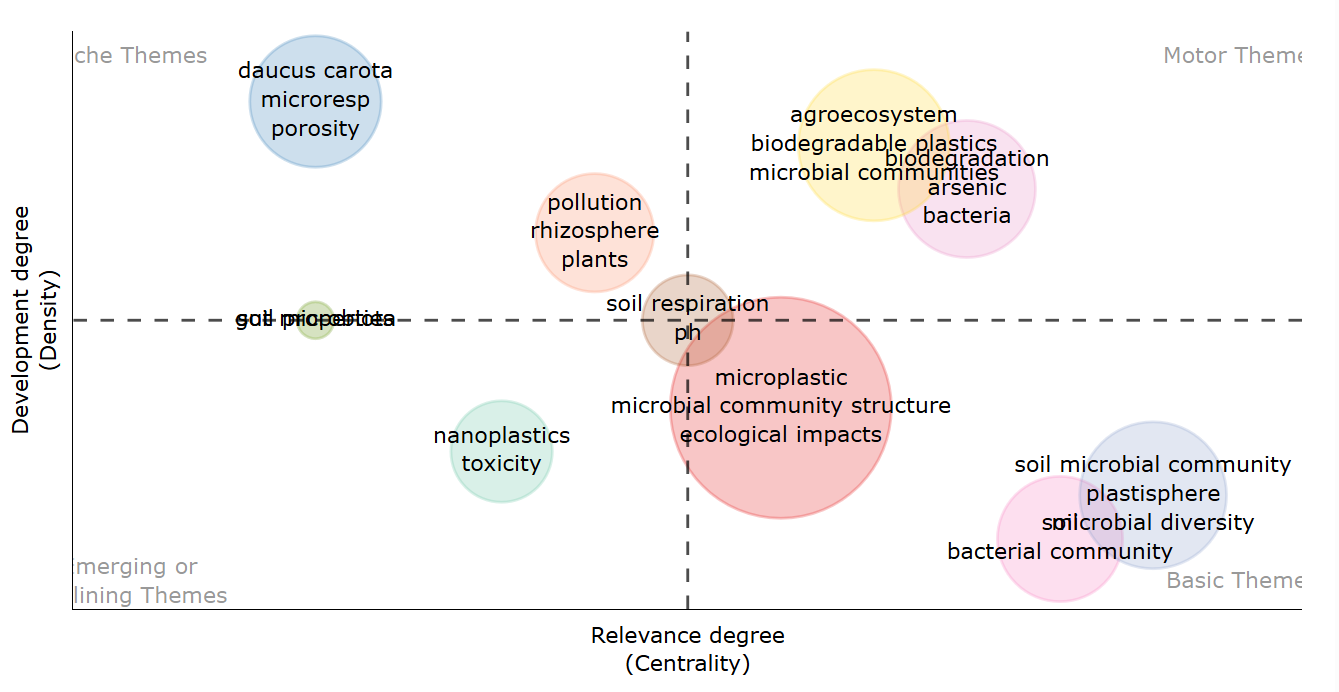


**(D)**


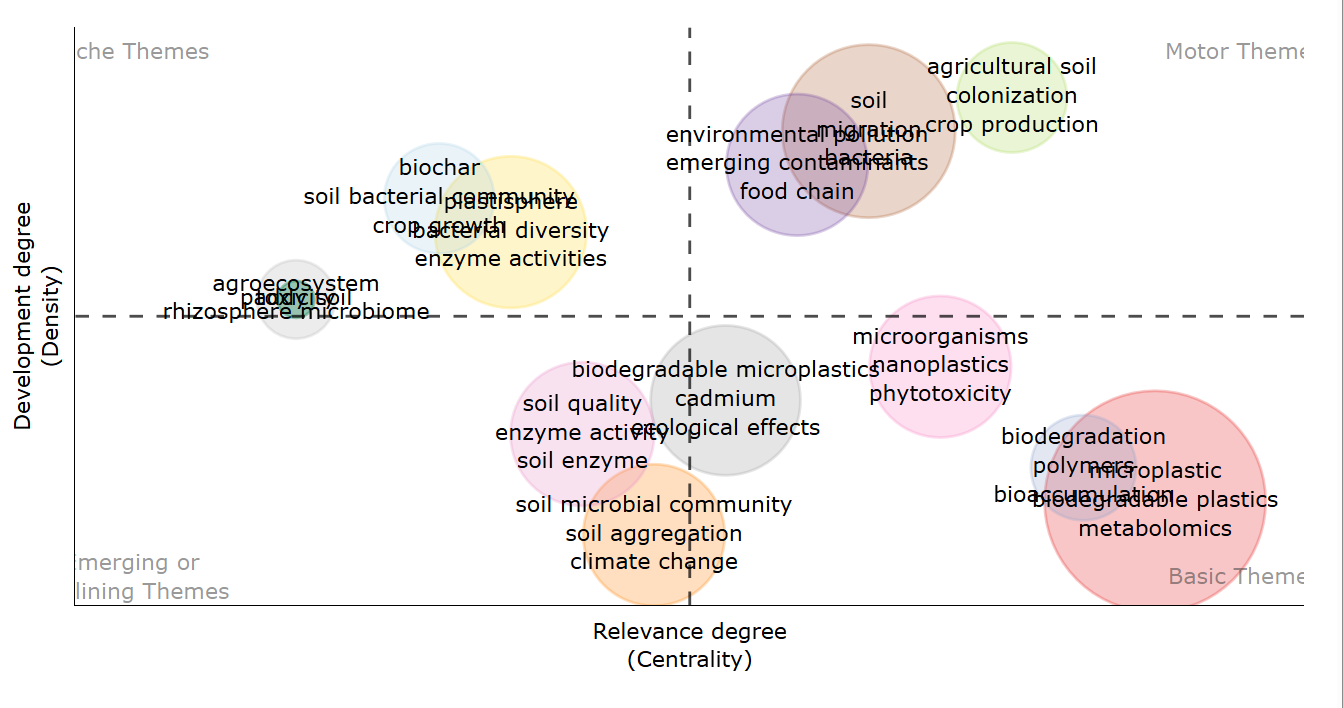

Supplement: Supplementary file 3 [file Presentation_1.zip › Supplementary Materials/Figure.docx]
